# Rapid alkalinization factor: function, regulation, and potential applications in agriculture

**DOI:** 10.1007/s44154-023-00093-2

**Published:** 2023-05-29

**Authors:** Ran Zhang, Peng-Tao Shi, Min Zhou, Huai-Zeng Liu, Xiao-Jing Xu, Wen-Ting Liu, Kun-Ming Chen

**Affiliations:** State Key Laboratory of Crop Stress Biology in Arid Area, College of Life Sciences, Northwest A&F University, Yangling, 712100 Shaanxi China

**Keywords:** RALF, Fertilization, Stress, Crops, Molecular regulatory network

## Abstract

Rapid alkalinization factor (RALF) is widespread throughout the plant kingdom and controls many aspects of plant life. Current studies on the regulatory mechanism underlying RALF function mainly focus on Arabidopsis, but little is known about the role of RALF in crop plants. Here, we systematically and comprehensively analyzed the relation between RALF family genes from five important crops and those in the model plant *Arabidopsis thaliana*. Simultaneously, we summarized the functions of RALFs in controlling growth and developmental behavior using conservative motifs as cues and predicted the regulatory role of RALFs in cereal crops. In conclusion, RALF has considerable application potential in improving crop yields and increasing economic benefits. Using gene editing technology or taking advantage of RALF as a hormone additive are effective way to amplify the role of RALF in crop plants.

**Supplementary Information:**

The online version contains supplementary material available at 10.1007/s44154-023-00093-2.

## Introduction

Plants have developed dynamic strategies to explore nutrients and adapt to the environment (Zhang, et al. 2020b). Many signaling molecules, hormones, small RNAs, and peptides are involved in regulating diverse biological processes (Araya, et al. 2014; Betti, et al. 2021; Gupta, et al. 2020), among which small peptides play vital roles in cell-to-cell communication due to their small size and diversity (Matsubayashi 2014).

Rapid alkalinization factor (RALF) is a cysteine-rich peptide directly translated from the small open reading frame (Tavormina, et al. 2015). RALFs are widely expressed in various plant tissues and organs and regulate reproduction, development, and response to external stimuli (Cao and Shi 2012). Since the first RALF was isolated from tobacco, the role of this cysteine-rich signaling molecule, named due to its ability to induce rapid alkalinization of the extracellular matrix in plant cells, has been elucidated gradually in plant cell communication (Pearce, et al. 2001).

The functions of RALFs are often dependent on their intercellular receptors. *Catharanthus roseus* receptor-like kinase 1-like proteins (*Cr*RLK1Ls) are a unique subfamily of plant receptor-like kinases (Boisson-Dernier, et al. 2011). These proteins play critical roles in controlling cell wall integrity and cell-to-cell communication and act as sensors for regulating development and environmental stimuli response in plants (Boisson-Dernier, et al. 2011; Xie, et al. 2022). Several studies have shown that the secreted peptide RALF, which exists in free unbound form outside the cell, could bind to the extracellular domain of FERONIA (FER), the famous member of the *Cr*RLK1Ls family (Campos, et al. 2018; Haruta, et al. 2014). After binding to FER, RALF initiates signal transduction events to orchestrate plant developmental processes and response to external stresses (Mingossi, et al. 2010; Zhao, et al. 2018).

RALFs are ubiquitous in terrestrial plants, and their earliest traces were found in bryophyte *Physcomitrium patens* (Campbell and Turner 2017). However, most studies on the functions of RALFs have been concentrated on the model plant Arabidopsis, and only a few studies have focused on its role and application in crops. In this review, we focus on the RALF family genes identified in the genomes of five significant crops, including rice (*Oryza sativa*), wheat (*Triticum aestivum*), maize (*Zea mays*), soybean (*Glycine max*), and rape (*Brassica rapa*). Based on a comprehensive analysis of the phylogenetic relationship, conserved motif features, and regulatory mechanisms of RALF genes, we reviewed the functions of RALFs in plant reproduction, development, and response to external stimuli, referring to their roles in *Arabidopsis thaliana*. Finally, we discuss the potential applications of RALFs in agriculture.

## RALF family genes in cereals and their phylogenetic relationship

RALF is widespread throughout the plant kingdom with the gene number varied widely among different species (Campbell and Turner 2017). Since the RALF family emerged in bryophytes, the scale of the RALF family expanded rapidly during angiosperm evolution (Campbell and Turner 2017). Repeated genome events and tandem repeat events are responsible for the part of RALF family expansion (Campbell and Turner 2017; Cao and Shi 2012; Olsen, et al. 2002). This is also consistent with the fact that whole-genome replication and fragment replication as the main way to drive the expansion of cysteine-rich peptides in most angiosperms (Liu, et al. 2017b). As the major receptors of RALFs in plants, the emergence timing and rapid expansion of the *Cr*RLK1L family immediately before the RALF family (Dievart, et al. 2020; Liu, et al. 2017a). It is likely that RALF evolved and expanded to match more complex and variable receptor families, thus responding to the needs of complex signaling pathways in plants.

**Fig. 1:**
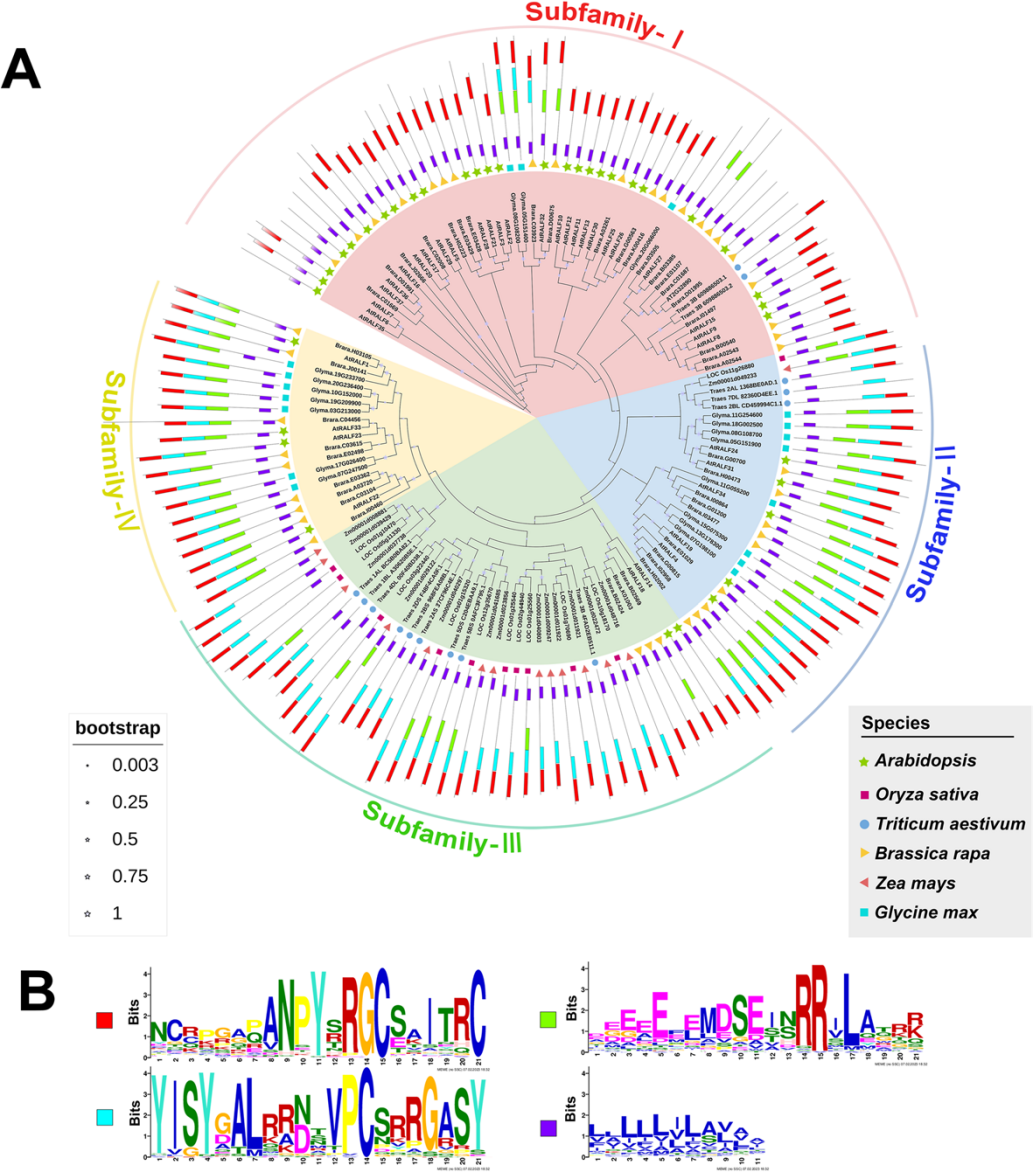
Phylogenetic analysis and conserved domain analysis of RALFs in five cereals and *Arabidopsis*. **A** The maximum-likelihood phylogenetic tree of RALF family members. The bootstrap value has been marked on the node by stars of different sizes. According to the motif differences and genetic relationship, RALFs can be divided into four subfamilies and denoted as Subfamilies -I, II, III and IV, respectively. **B** The logos of conserved motif sequences. The logos of conserved domain sequences were obtained from the MEME suite website (https://meme-suite.org/meme/). The bit score represents information content of each position in the amino acid sequence. The conserved motif was marked in different colors and displayed on the periphery of the phylogenetic tree. The evolutionary tree is modified with ITOL (https://itol.embl.de/)

The RALF family has 38 genes in *Arabidopsis thaliana*. However, AT4G14020, which lacks a signal peptide, was recently excluded from the RALF family (Abarca, et al. 2021). Although the functions of RALFs have been widely studied in Arabidopsis, not much RALFs have been identified in cereal species such as wheat, rice, and maize, etc. To get insights into the roles of RALFs in plants. Here, we performed research on the genomes of five important cereal crops based on the sequence feature of RALFs in Arabidopsis, and total 96 RALF genes were identified in these crops with 11, 11, 42, 14, and 18 members in rice, wheat, rape, maize, and soybean, respectively (Table S1). Similarly, we also excluded the non-transmembrane signal peptide RALFs as the real RALFs of the five cereal crops. Running all RALF proteins on the MEME tool (https://meme-suite.org/meme/), we detected four conserved motifs in amino acid sequences of the cereal RALFs: the conserved cysteine residues at the C-terminus, the YIXY motif, the RRXL shear site, and the leucine-rich N-segment region (Fig. 1B).

To clarify the phylogenetic relationship of the cereal RALFs, a phylogenetic tree was established based on their amino acid sequences with the members of the RALFs in *Arabidopsis thaliana* as the references using MEGAX software (http://www.megasoftware.net). As shown in Fig. 1A, the cereal crop RALFs can be divided into four subfamilies. Most of the RALFs classified as Subfamily I, which did not have the YIXY motif and the RRXL shear site recognized by site-1 protease (S1P), while most of the RALFs in Subfamily III only lacked the RRXL site. The most widely studied RALFs belong to Subfamilies II and IV containing the four typical motif domains. The diversity of cereal RALFs indicates their functional divergence as their homologs in Arabidopsis.

## Functions of RALFs

### Predicted functions of RALFs in crops based on their conserved motifs

Members of the RALF family have low amino acid sequence specificity, but conserved structural features are necessary for RALF function. Although studies on RALFs in crops are rare, these conservative motifs provide critical cues for understanding their roles in crop growth and development.

#### S1P site

The precursor proteins of RALFs generally have a signal peptide at the N-terminal, processed by signal peptidases during translocation. The sequences of RALF precursors also contain two basic sites, RRXL or RXLX, located upstream of the active center of the precursor proteins. In Arabidopsis, the dibasic sites can be recognized and cleaved by subtilisin-like proteinases, *At*S1P (Matos, et al. 2008; Srivastava, et al. 2009). The plants overexpressing *At*RALF23 with or without S1P site mutation showed different phenotypes, indicating that the pro-RALF with dibasic sites needs to be processed correctly to perform biological functions (Srivastava, et al. 2009). The dibasic recognition cleavage site is also present in the peptide hormones such as phytosulfokine, CLAVATA3, and *Z. mays* immune signaling peptide1, which can be hydrolyzed and processed by subtilisin-like proteinases (Berger, et al. 2000; Yang, et al. 2001; Ziemann, et al. 2018).

However, the number of RALFs with S1P sites is essentially the same as that of RALFs lacking S1P sites. In *P. patens*, the hydrolytic cleavage of RALF protein is required for its function (Ginanjar, et al. 2022). These results imply that the functional differentiation of RALF peptides started in primitive land plants. In addition, the functionally differentiated RALFs mediate opposite phenotypes via FER; therefore, the presence or absence of S1P sites is a critical characterization of RALF peptides (Stegmann, et al. 2017a). In a few RALF members, another dibasic recognition cleavage occurs at the C-terminus of the RRXL/RXLX recognition site with high frequency (Olsen, et al. 2002). However, whether they also serve as shear recognition sites in the degradation of inactivated mature RALFs is still unknown.

#### N-terminal YIXY motif

A unique YISY motif, a form of YIXY, was found at amino acids position 5–8 at the N-terminal of the RALF. Along with leucine at position 11, the YISY motif formed a significant spatial configuration for binding to downstream receptors. The absence of this spatial conformation inhibits RALF-induced alkalinization of the extracellular matrix and reduces root length inhibition (Pearce, et al. 2010; Xiao, et al. 2019). The short linear binding motifs determine the extent of RALFs binding to their downstream receptors, and therefore, evolutionary changes in the binding motifs represent the dynamicity of protein interactions.

The YIXY motif is necessary for the biological activity of RALFs (Pearce, et al. 2010), but the binding site of RALF peptides to their corresponding receptors is not necessarily a YIXY motif (Moussu, et al. 2020). For example, the recognition binding of RALF4 to BUDDHA'S PAPER SEAL/ANXUR (BUPS/ANX) receptor complex is not dependent on YIGY. The binding of RALF4 to LORELEI (LRE)-like glycolphosphatidylinositol-anchored protein (GPI-AP) 3 (LLG3) is achieved mainly through the region between the second and fourth cysteine residues at the C-terminus of the RALF (Ge, et al. 2019). However, RALF4 requires the YIGY motif to maintain pollen tube integrity (Ge, et al. 2019). In addition, the interaction of RALF23 or RALF1 with FER depends on the C-terminal region of RALFs (Liu, et al. 2018; Xiao, et al. 2019).

#### C-terminal cysteine residues

Four cysteine residues are present at the C- terminal of most RALFs, and these residues were found to be involved in disulfide bond formation and proper folding of the RALF peptide (Abarca, et al. 2021). The C-terminus of RALF23 can enhance the overall stability of the complex by binding to FER (Xiao, et al. 2019). In addition, polar and charged amino acids present at the C-terminus of RALF peptides can influence the tertiary structure of proteins, and the extension of these sequences may also determine the specificity of RALFs binding to different receptors (Olsen, et al. 2002). Recent studies reported that RALFs from nematodes and *Fusarium oxysporum* lack the first disulfide bond but have the second disulfide bond at the end of their C-terminus, as in plants. This finding implies that the second disulfide bond is evolutionarily more conserved and functional than the first disulfide bond (Thynne, et al. 2017; Zhang, et al. 2020a). The second disulfide bond at the C-terminus of *At*RALF1 is indispensable for the negative regulation of RALF in plant immune responses to biotic stress (Zhang, et al. 2020a).

### RALFs can be sensed by receptors and induce the formation of receptor complexes

RALFs perform biological functions by binding to their receptors on the cell membrane (Du, et al. 2016; Ge, et al. 2019; Gonneau, et al. 2018; Haruta, et al. 2014; Zhong, et al. 2022). The molecular mechanisms underlying RALF recognition by *Cr*RLK1L receptors and the subsequent receptor activation have been described successfully (Moussu, et al. 2020; Stegmann, et al. 2017a; Xiao, et al. 2019).

RALF8 is highly disordered in solution except for an ordered ring consisting of disulfide bonds. RALF is likely induced to form the aligned structure only upon binding to the receptor (Frederick, et al. 2019). For the corresponding receptor *Cr*RLK1Ls, a deep cleft between the two extracellular malectin-like domains arranged by highly conserved aromatic and polar amino acid residues implies that this may be a potential RALF-binding site (Du, et al. 2018; Moussu, et al. 2018).

As the researchers analyzed the structure of the ligand-receptor complexes formed by RALF and its binding receptors, they reported that RALF achieved the formation of ligand-induced receptor complexes through different mechanisms (Moussu, et al. 2020; Xiao, et al. 2019). Various proteins were found as co-receptors of *Cr*RLK1Ls, involved in the recognition and binding of RALFs (Ge, et al. 2019; Stegmann, et al. 2017b; Xiao, et al. 2019). The protein LRE, and its homolog LLG assist in the structural stabilization and modification of FER, thereby controlling the location and timing of FER transduction signals (Li, et al. 2015; Liu, et al. 2016; Noble, et al. 2022; Xiao, et al. 2019). The protein LLG1 acts as a co-receptor for FER and assists in transporting FER from the endoplasmic reticulum to the cell membrane surface (Li, et al. 2015). The presence of RALF23 promotes the formation of the LLG–FER receptor complex, as confirmed by analytical ultra-centrifugation and co-immunoprecipitation assays (Xiao, et al. 2019). BAK1 participates in the immune response as a co-receptor of leucine repeat receptor kinases (LRKs) (Couto and Zipfel 2016; Roux, et al. 2011; Schulze, et al. 2010; Wang, et al. 2021), and RALF binding enhances the recruitment of immune recognition complexes to BAK1 (Stegmann, et al. 2017b).

Analysis of the crystal structure of the RALF-receptor complex reveals that different motifs in RALFs play critical roles in binding to the corresponding receptors. The N-terminus of RALF23 formed an α-helix to penetrate the groove on the surface of the LLG2–FER complex, and the YISY motif at the N-terminal of RALF23 interacted with LLG2 through a hydrophobic polar structure. The C-terminus of RALF23 enhanced the stability of the complex by binding to FER (Xiao, et al. 2019). Covalent cross-linking and mass spectrometry also demonstrated that the extracellular domain of FER binds to the conserved C-terminus of RALF1, and the deletion of the YISY motif in RALF1(Δ2–8) also resulted in a 50% reduction in binding efficiency to FER (Liu, et al. 2018). This finding demonstrates that the YISY motif in the N-terminal of RALF is involved in the formation of α-helical structures, which is critical for the binding of LLGs and FER (Liu, et al. 2018; Xiao, et al. 2019). Leucine-rich repeat extension proteins (LRXs) also act as a sensing target for RALF4/19 in maintaining cell wall integrity (Mecchia, et al. 2017). Resolution of the crystal structures of RALF4–LRX2 and RALF4–LRX8 revealed that disulfide bond-induced correct folding of RALF4 greatly enhanced its ability to bind to the core LRR domain of LRXs. The N-terminal 63Y, 64I, 66Y of RALF4 is located within the binding region but is not a critical factor in the high-affinity binding of RALF4 to LRX. The correct folding structure oriented by the internal disulfide bond of RALF4 is the key to tight binding (Moussu, et al. 2020), consistent with the results of physiological experiments (Ge, et al. 2019; Moussu, et al. 2020).

These results provide the basis for elucidating the role of dynamic assembly between receptors in signal transduction (Liu, et al. 2018; Moussu, et al. 2020; Xiao, et al. 2019). Recently, a few *Cr*RLK1L receptor complexes have been identified as RALF receptors to play a role in the regulation of plant development, such as receptors FER/ANJEA (ANJ)/ HERCULES RECEPTOR KINASE 1(HERK1) and BUPS1/2–ANX1/2 (Ge, et al. 2017; Ge, et al. 2019; Liu, et al. 2021; Zhong, et al. 2022). The possibility of RALF peptide inducing multiple *Cr*RLK1L receptors to form a supramolecular receptor complex is to be investigated.

### Role of typical RALFs with conserved motifs in plant reproduction and development

These typical RALF peptides with four conserved motifs are recognized by receptors and trigger a series of relatively redundant functional responses in the root system and reproductive tissues. We summarize the regulatory functions of Arabidopsis typical members of the RALF family on plant reproduction and development (Table 1, Subfamily-II, IV), and summarize the roles of RALF members in other crops (Table 2).

**Table 1:** Members of the Arabidopsis RALF family

	**Name**	**Gene ID**	**Receptors**	**Location**	**Regulation**	**References**
Subfamily-I	*At*RALF35	AT1G60913	FER	Root system	Root growth inhibition	(Abarca, et al. 2021)
			Unknown		Increase elf18-induced ROS	(Abarca, et al. 2021)
	*At*RALF6	AT1G60625	FER/ANJ/HERK1	Pollen and pollen tubes	Controls the polytubey block	(Zhong, et al. 2022)
			FER		Increase elf18-induced ROS	(Abarca, et al. 2021)
			FER	Root system	Root growth inhibition	(Abarca, et al. 2021)
	*At*RALF7	AT1G60815	FER/ANJ/HERK1	Pollen and pollen tubes	Controls the polytubey block	(Zhong, et al. 2022)
			FER		Increase elf18-induced ROS	(Abarca, et al. 2021)
			FER	Root system	Root growth inhibition, extracellular alkalinizing activity	(Abarca, et al. 2021)
	*At*RALF37	AT2G32788	FER/ANJ/HERK1	Pollen and pollen tubes	Controls the polytubey block	(Zhong, et al. 2022)
	*At*RALF36	AT2G32785	FER/ANJ/HERK1	Pollen and pollen tubes	Controls the polytubey block	(Zhong, et al. 2022)
			Unknown	Root system	Root growth inhibition, extracellular alkalinizing activity	(Abarca, et al. 2021; Gjetting, et al. 2020)
			FER		Increase elf18-induced ROS	(Abarca, et al. 2021)
	*At*RALF16	AT2G32835	FER/ANJ/HERK1	Pollen and pollen tubes	Controls the polytubey block	(Zhong, et al. 2022)
			FER		Increase elf18-induced ROS	(Abarca, et al. 2021)
			FER	Root system	Root growth inhibition, extracellular alkalinizing activity	(Abarca, et al. 2021)
	*At*RALF20	AT2G34825	FER	Root system	Root growth inhibition	(Abarca, et al. 2021)
			FER		Increase elf18-induced ROS	(Abarca, et al. 2021)
	*At*RALF17	AT2G32885	FER		Increase elf18-induced ROS	(Stegmann, et al. 2017b)
			FER	Root system	Root growth inhibition	(Abarca, et al. 2021)
	*At*RALF29	AT4G11653	Unknown		Increase elf18-induced ROS	(Abarca, et al. 2021)
	*At*RALF5	AT1G35467				
	*At*RALF28	AT4G11510				
	*At*RALF21	AT3G04735	FER	Root system	Root growth inhibition	(Abarca, et al. 2021)
	*At*RALF3	AT1G23147				
Subfamily-I	*At*RALF2	AT1G23145				
	*At*RALF32	AT4G14010	FER		Increase elf18-induced ROS	(Stegmann, et al. 2017b)
			Unknown	Root system	Root growth inhibition	(Abarca, et al. 2021)
	*At*RALF10	AT2G19020	FER		Increase elf18-induced ROS	(Abarca, et al. 2021)
	*At*RALF12	AT2G19040				
	*At*RALF11	AT2G19030				
	*At*RALF13	AT2G19045	FER		Increase elf18-induced ROS	(Abarca, et al. 2021)
	*At*RALF30	AT4G13075	FER		Increase elf18-induced ROS	(Abarca, et al. 2021)
	*At*RALF25	AT3G25165				
	*At*RALF26	AT3G25170				
	*At*RALF27	AT3G29780				
	*At*RALF15	AT2G22055	FER	Root system	Root growth inhibition	(Abarca, et al. 2021)
	*At*RALF9	AT1G61566	FER		Increase elf18-induced ROS	(Abarca, et al. 2021)
			FER	Root system	Root growth inhibition	(Abarca, et al. 2021)
	*At*RALF8	AT1G61563	Unknown		Negative regulation of tolerance to drought and nematode infection	(Atkinson, et al. 2013)
			FER	Root system	Root growth inhibition	(Abarca, et al. 2021; Frederick, et al. 2019)
			FER		Increase elf18-induced ROS	(Abarca, et al. 2021)
	*At*RALF24	AT3G23805	FER	Root system	Root growth inhibition, extracellular alkalinizing activity	(Abarca, et al. 2021; Morato do Canto, et al. 2014)
Subfamily-II			Unknown		Increase elf18-induced ROS	(Abarca, et al. 2021)
	*At*RALF31	AT4G13950	Unknown	Root system	Root growth inhibition, extracellular alkalinizing activity	(Morato do Canto, et al. 2014)
			Unknown		Increase elf18-induced ROS	(Abarca, et al. 2021)
	*At*RALF34	AT5G67070	THE1	Root system	Primary lateral root development	(Gonneau, et al. 2018)
			FER		Inhibit elf18-induced ROS	(Stegmann, et al. 2017b)
			BUPS1/2—ANX1/2	Ovules	Induction of pollen tube rupture and sperm release	(Ge, et al. 2017)
Subfamily-II			Unknown	Root system	Root growth inhibition, extracellular alkalinizing activity	(Abarca, et al. 2021; Morato do Canto, et al.^ 2014^)
	*At*RALF19	AT2G33775	BUPSs/ANXs/LLGs	Pollen tube	Maintaining the integrity of the pollen tube	(Ge, et al. 2017; Ge, et al. 2019)
			FER	Root system	Root growth inhibition, extracellular alkalinizing activity	(Abarca, et al. 2021; Morato do Canto, et al. 2014)
			LRXs	Pollen tube	Maintaining the integrity of the pollen tube	(Mecchia, et al. 2017)
			FER/LRE	Synergids	Triggered calcium ion oscillation	(Gao, et al. 2022)
			FER		Inhibit elf18-induced ROS	(Abarca, et al. 2021)
	*At*RALF4	AT1G28270	BUPSs/ANXs/LLGs	Pollen tube	Maintaining the integrity of the pollen tube	(Ge, et al. 2017; Ge, et al. 2019)
			Unknown	Root system	No root growth inhibition, no extracellular alkalinizing activity	(Morato do Canto, et al. 2014)
			LRXs	Pollen tube	Maintaining the integrity of the pollen tube	(Mecchia, et al. 2017)
			FER/LRE	Synergids	Triggered calcium ion oscillation	(Gao, et al. 2022)
			Non-FER		Inhibit elf18-induced ROS	(Abarca, et al. 2021)
Subfamily-IV	*At*RALF22	AT3G05490	FER	Root system	Root growth inhibition, extracellular alkalinizing activity	(Gonneau, et al. 2018; Morato do Canto, et al. 2014)
			FER		Negative regulation of salt tolerance	(Zhao, et al. 2018)
			FER		Inhibit elf18-induced ROS	(Abarca, et al. 2021)
	*At*RALF23	AT3G16570	FER	Root system	Root growth inhibition, extracellular alkalinizing activity	(Abarca, et al. 2021; Gonneau, et al. 2018)
			FER		Inhibit elf18-induced ROS, negative regulation of plant immunity through the accumulation of MYC	(Guo, et al. 2018; Stegmann, et al. 2017b)
			FER		Promotion of Pseudomonas colonization	(Song, et al. 2021)
			ANJ/FER	Pollen-stigma	Involvement in affinity pollination mechanisms	(Liu, et al. 2021)
			FER		Negative regulation of salt tolerance	(Zhao, et al. 2018)
	*At*RALF33	AT4G15800	FER	Root system	Root growth inhibition, extracellular alkalinizing activity	(Gjetting, et al. 2020; Gonneau, et al. 2018)
			FER		Inhibit elf18-induced ROS	(Stegmann, et al. 2017b)
			FER		Promotion of Pseudomonas colonization	(Song, et al. 2021)
			ANJ/FER	Pollen-stigma	Involvement in affinity pollination mechanisms	(Liu, et al. 2021)
	*At*RALF1	AT1G02900	FER	Root system	Root growth inhibition, extracellular alkalinizing activity	(Chen, et al. 2016; Haruta, et al. 2014)
			BAK1	Root system	Root growth inhibition	(Dressano, et al. 2017)
			FER		Inhibit elf18-induced ROS	(Abarca, et al. 2021)
			FER		Affect the flowering time	(Wang, et al. 2020a)
			FER		Increasing salt toxicity by increasing ion concentration	(Yu and Assmann 2018)
Subfamily-III	*At*RALF18	AT2G33130				
	*At*RALF14	AT2G20660				

Corresponding receptors and functions of Arabidopsis RALF family members in different tissues

**Table 2:** Members of the RALF family in the crops

Species	Gene ID	Name	Homologous Arabidopsis RALF	Regulation	References
*Strawberry*	FvH4_2g25351	*Fa*RALF-33-like	*At*RALF33	Affect the susceptibility to *C. acutatum*	(Merino, et al. 2019)
*Glycine max*	Glyma.03G213000	*Gm*RALF4	*At*RALF23	Immune response to *F. oxysporum*	(Liu, et al. 2022b)
*Glycine max*	Glyma.19G209900	*Gm*RALF24	*At*RALF23	Immune response to *F. oxysporum*	(Liu, et al. 2022b)
*Brassica napus*	Brara.A03720	*Bn*RALF10	*At*RALF22	Immunity response to *S. sclerotiorum*	(He, et al. 2022)
*Saccharum hybrid cultivar*	CA182793	*Sac*RALF1		Inhibition of tissue expansion	(Mingossi, et al. 2010)
*Chenopodium quinoa*	AUR62000768	*Cq*RALF15	*At*RALF22	Involved in salt stress response	(Jiang, et al. 2022)
*Taraxacum koksaghyz*	AEE27494.1	*Tk*RALFL1	*At*RALF1	Affect root phenotype	(Wieghaus, et al. 2019)
*Oryza sativa*	LOC_Os01g25560	*Os*RALFL7		Involved in immunity response	(Wang, et al. 2020c)
*Oryza sativa*	LOC_Os02g44940	*Os*RALFL8		Involved in immunity response	(Wang, et al. 2020c)
*Hevea brasiliensis*	scaffold0558_759063	*Hb*RALF3		Regulation of pH homeostasis in Hevea latex	(Sui, et al. 2023)
*Hevea brasiliensis*	scaffold0625_679353	*Hb*RALF19		Regulation of pH homeostasis in Hevea latex	(Sui, et al. 2023)
*G. klotzschianum*		RALFL33		Response to cold and salt stress	(Xu, et al. 2020)
*Solanum Lycopersicum*	SGN-U324197	*SlP*RALF	*At*RALF4	Specific inhibition of pollen tube elongation	(Covey, et al. 2010)
*Pyrus bretschneideri*	Pbr020387.1	*Pbr*RALF2		Inhibition of pollen tube elongation	(Kou, et al. 2021)
*Physcomitrium patens*	Pp3c6_7200V3	*Pp*RALF2		Promote protonema tip growth, and response to phytopathogens	(Ginanjar, et al. 2022; Mamaeva, et al. 2023)
*Physcomitrium patens*	Pp3c25_4180V3	*Pp*RALF3		Response to abiotic stress factors and phytopathogens	(Mamaeva, et al. 2023)
*Medicago truncatula*	MtC90970	*Mt*RALFL1		Affect rhizobial infection and nodulation	(Combier, et al. 2008)
*Solanum chacoense*	AY422826	*Sc*RALF3	*At*RALF34	Affect the development of gametophyte	(Chevalier, et al. 2013; Loubert-Hudon, et al. 2020)

The members of the RALF family in the crops. The function and gene ID of RALF in other crops were listed. The RALF gene homologous to Arabidopsis RALF is also annotated

#### RALFs play a precise molecular gating role in different stages of plant reproduction

**Fig. 2:**
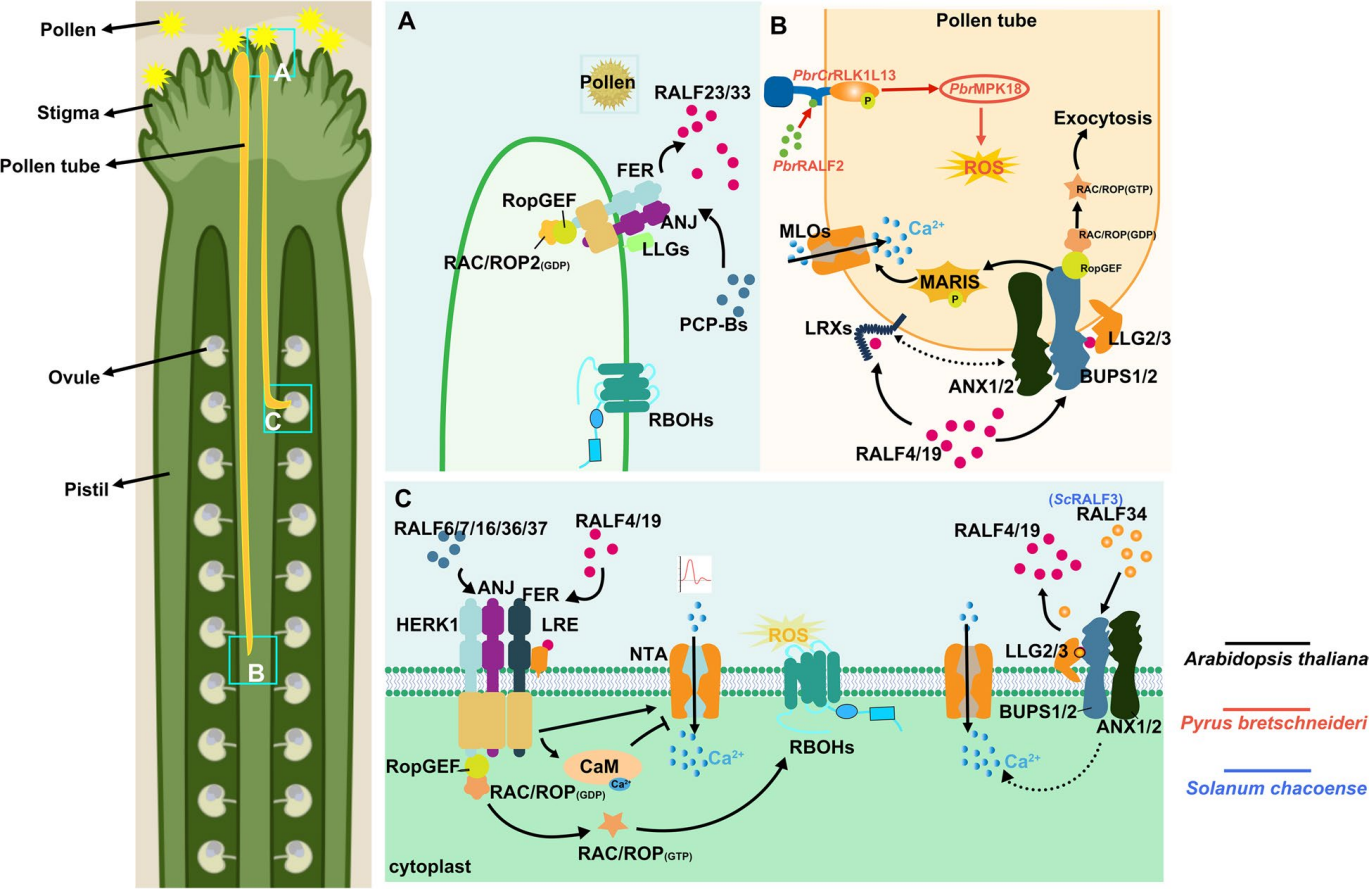
RALFs play a precise molecular gating role in different stages of plant reproduction. **A** On the pollen-stigma surface, RALF23/33 competes with PCP-Bs to bind to the receptor complex FER/ANJ/LLGs, regulates ROS production through the downstream GTPase activation pathway, thus affects pollen hydration (Liu, et al. 2021). **B** At the pollen tube extension process, RALF4/19 activates GTPase and the cytoplasmic receptor-like kinase MARIS through the receptor complex. Pollen tube integrity is maintained by maintaining exocytosis at the pollen tube tip and Ca^2+^ concentration gradient (Boisson-Dernier, et al. 2013; Gao, et al. 2023; Ge, et al. 2017). In pear, *Pbr*RALF2 binds to the ectodomain of *Pbr*CrRLK1L13 and induces phosphorylation of *Pbr*CrRLK1L13, which induces ROS production through downstream *Pbr*MPK18, and excess ROS negatively regulates pollen tube elongation (Kou, et al. 2021). **C** At the pollen tube-synergids interface, RALF4/19 sensed by the receptor complex recruit membrane channel protein NTA, trigger Ca^2+^ oscillation and participate in the process of pollen tube reception. CaM has an inhibitory feedback mechanism on the activity of NTA, thereby preventing the excessive Ca^2+^ influx activated by RALFs (Gao, et al. 2022). The receptor complex induces the rupture of pollen tubes reaching female gametophytes through activation of GTPase-RBOHs resulting in the increasing of ROS (Duan, et al. 2014). The substitution of RALF34 for RALF4/19 induced pollen tube rupture (Ge, et al. 2017). RALF6/7/16/36/37 maintain high-efficiency double fertilization by binding to the FER/ANJ/HERK1 receptor complex (Zhong, et al. 2022). Annotated Arabidopsis RALF homologs in other species

The unique double fertilization process in plants affects the following five major important events: (1) pollen–stigma recognition, (2) pollen tube germination and polar growth in the style, (3) pollen tubes extending toward micropyle and entering the embryo sac, (4) rupture of the pollen tube tip and release of sperm and other contents after the pollen tube reaches the embryo sac, and (5) recognition and fusion of sperm cells with female gametes (Dresselhaus, et al. 2016). Timely signal exchange during this process is a prerequisite for the effective fusion of male and female gametes. In this finely regulated process, RALF plays a crucial role in female–male signal communication (Fig. 2) (Gao, et al. 2023; Ge, et al. 2017; Liu, et al. 2021; Mecchia, et al. 2017; Wang, et al. 2020a).

The ruptured anthers release pollen onto the moist female stigma. Subsequently, pollen hydration promotes pollen tube germination and elongation (Dresselhaus, et al. 2016). Liu, et al. (2021) reported that RALF23/33 inhibited pollen hydration by activating the downstream RAC/ROP-NADPH oxidase to induce reactive oxygen species (ROS) generation through the ANJ –FER receptor kinase complex in the stigma. On the contrary, the POLLEN COAT PROTEIN B (PCP-B) peptide competed with RALFs to bind to FER, and the predominance of PCP-B inhibited ROS generation in the stigma after pollination, promoting pollen hydration and initiating pollen tube germination.

After breaking through the papilla cell barrier, pollen tubes continuously elongate in the style tissue (Iwano, et al. 2014). Several members of the *Cr*RLK1L family have been implicated in regulating cell elongation in the pollen tube (Boisson-Dernier, et al. 2013; Boisson-Dernier, et al. 2009; Zhou, et al. 2021; Zhu, et al. 2018), whereas RALFs maintain pollen tube integrity by activating the members of the *Cr*RLK1L family (Ge, et al. 2017; Zhou, et al. 2021). ANXUR1 and ANXUR2, localized on the plasma membrane of the pollen tube tip, inhibit the rupture of pollen tubes that fail to reach female gametophyte by activating exocytotic secretion (Boisson-Dernier, et al. 2013; Boisson-Dernier, et al. 2009). The dramatic mechanical pressure at the pollen tube tip in the face of penetrating style tissue requires BUPS1-induced cell wall strengthening to avoid rupturing of the tube tip (Zhou, et al. 2021; Zhu, et al. 2018). RALF4/19 binds to the receptors BUPS1/2 and ANX1/2 located on the apical plasma membrane of the pollen tube via an autocrine signaling pathway, thereby maintaining pollen tube integrity during polar elongation of the pollen tube (Ge, et al. 2017). Low-level expression of *LLG2* and *LLG3* also caused defects in pollen tube development (Feng, et al. 2019; Ge, et al. 2019). The proteins LLG2/3 serve as chaperones for BUPS1/2 and ANX1/2, assisting their transport to the plasma membrane at pollen tube tips (Feng, et al. 2019) and also act as co-receptors of the BUP1/2–ANX1/2 receptor complexes, enhancing the reception of RALF4/19 signaling (Ge, et al. 2019). RALF4 induces ROS through the receptor complex involved in pollen tube growth and cell wall strengthening (Feng, et al. 2019). The stress-induced release of RALF4/19 from the penetrating style tissue further amplifies plant response to mechanical stress (Zhou, et al. 2021).

RALF4/19 is also dependent on pollen tube expressed LRX protein to inhibit the accumulation of acidic pectin at the pollen tube tip, and conversely, RALF4/19 promotes the increase of callus, which hardens the cell wall. RALF4 sensing is reduced significantly with the LRX8/9/10/11 mutation (Mecchia, et al. 2017). It needs to be explored whether pollen tube-expressed LRX can form supramolecular heterodimers with LLG/BUPS/ANX receptor complex or whether there is a relation between LRX and the receptor complex.

The pollen tube extends to the surrounding of the embryo sac and continues to elongate around the filiform apparatus area of synergids toward the gamete fusion site (Denninger, et al. 2014). The FER–LRE receptor complex generates ROS in a calcium (Ca^2+^)-dependent manner via ROPGEF-RAC/ROP in the filiform apparatus area (Capron, et al. 2008; Duan, et al. 2014; Liu, et al. 2016). High levels of ROS induce the apical rupture of pollen tubes that reach the synergids on the side of the micropyle, releasing sperm and other contents (Duan, et al. 2014). RALF4/19 activates the mechanism underlying pollen tube acceptance through the receptor FER/LRE on the surface of synergids (Gao, et al. 2022). HERK1 and ANJ, as members of the *Cr*RLK1L family, act synergically with FER/LRE in the filiform apparatus area of synergids, participating as female determinants to accomplish pollen tube reception and peptide sensing (Galindo-Trigo, et al. 2020). RALF34, located in the ovule, sends paracrine signals to the pollen tube and competes with RALF4/19 to bind to the BUP1/2–ANX1/2 receptor complex, thus triggering pollen tube apical rupture to release contents when it is dominant (Ge, et al. 2017). RALF4/19 and RALF34 antagonistically regulate the cell wall integrity of the pollen tube and prevent premature rupture. The receptor FER has a dual role in ensuring sperm transmission and preventing multiple sperm fertilization. Decrease in de-esterified pectin and nitric oxide (NO) content in the filiform apparatus of *fer*, an Arabidopsis mutant of FER, mitigating the inhibitory effects on LUREs, which are pollen tube inducers, eventually triggering the penetration of multiple pollen tubes into ovules (Duan, et al. 2014, 2020; Okuda, et al. 2009). Several RALFs lacking S1P sites also show the capability of preventing multiple pollen tubes from targeting the ovule (Zhong, et al. 2022). However, whether RALF peptides are related to LUREs, NO, and other molecules is unknown.

The Ca^2+^ signaling is involved in almost all steps of angiosperm double fertilization (Denninger, et al. 2014; Duan, et al. 2014; Ngo, et al. 2014; Schiøtt, et al. 2004). In addition, recent studies have implicated Ca^2+^ signals in maintaining the integrity of the pollen tube wall (Gao, et al. 2023). Mutations in MARIS, a receptor-like cytoplasmic kinase downstream of NADPH oxidase, result in the rupture of pollen tube tips (Boisson-Dernier, et al. 2015). RALF4/19 was found to bind to receptor complexes and activate Ca^2+^-permeable channel proteins MILDEW RESISTANCE LOCUS O (MLO)1/5/9/15 via the cytoplasmic-like receptor kinase MARIS, maintaining Ca^2+^ gradient in the cytosol of pollen tube tips and the integrity of pollen tubes (Gao, et al. 2023). MARIS is located downstream of respiratory burst oxidase homologs (RBOHs), which produce stable ROS that may regulate Ca^2+^ gradient homeostasis via RALF-activated receptors, thus maintaining pollen tube stability (Boisson-Dernier, et al. 2015; Boisson-Dernier, et al. 2013). The reception of sperm by the female gametophyte depends on highly coordinated FER-mediated Ca^2+^ signaling (Ngo, et al. 2014). Pollen tube contact with the ovule triggers Ca^2+^ oscillations that depend on the calmodulin-gated Ca^2+^ channel, NORTIA (NTA). RALF4/19 is sensed by the FER/LRE receptor complex and drives the recruitment of the membrane channel protein NTA, which triggers Ca^2+^oscillations on the synergid cell side and thus participates in pollen tube reception (Gao, et al. 2022). The increase in ROS generation in the filiform apparatus area may weaken the intensity of the cell wall of the pollen tube, and Ca^2+^ oscillations in the cytoplasm of synergids may eventually induce the rupture of the pollen tube tip (Duan, et al. 2014; Gao, et al. 2022). However, the diversity and complexity of Ca^2+^-regulated cellular responses increase our difficulty associating RALF-regulated calcium signals with other signals.

Similar to RALF in Arabidopsis, RALF in crops also plays an irreplaceable role in plant reproduction, and the exploration of RALF in crops further complements the analysis of plant double fertilization. *Solanum lycopersicum* RALF (*SlP*RALF), can specifically inhibit the elongation of pollen tubes in vitro, and the inhibitory effect of *SlP*RALF on pollen tubes is eliminated until the pollen tube is 40–60 μm long. The node timing of male germ unit entry into pollen tubes may be related to *SlP*RALF inhibition removal (Covey, et al. 2010). Recent studies have found that RALF also acts as a negative regulator of pollen tube elongation in pears, *Pyrus bretschneideri*. The *Pbr*RALF2–*Pbr*CrRLK1L13–*Pbr*MPK18 module uniquely inhibits pollen tube growth by producing excessive ROS (Kou, et al. 2021). The inhibitory effect of RALFs on the development of pollen tubes in crops may be a way for plants to self-regulate the stable growth of pollen tubes in response to the external environment. *Sc*RALF3 in *Solanum chacoense* shows us another potential function of the RALF family in plant reproduction. RALFs may act as a communication peptide between sporophytes and gametophytes and participate in the induction of maturation of male or female gametophytes. *scralf3* RNAi lines exhibit delay or arrest in embryo sac development during megagametogenesis. Pollen development is impaired in *scralf3* RNAi lines during mitosis I, thus showing a lower setting percentage (Chevalier, et al. 2013; Loubert-Hudon, et al. 2020). *At*RALF34 is crucial for triggering female–male signal communication (Ge, et al. 2017). However, whether its homolog *Sc*RALF3 also promotes the rupture of pollen tubes reaching the ovule remains to be investigated.

#### RALFs act as endogenous signaling peptides to regulate root development by controlling plant cell expansion

**Fig. 3:**
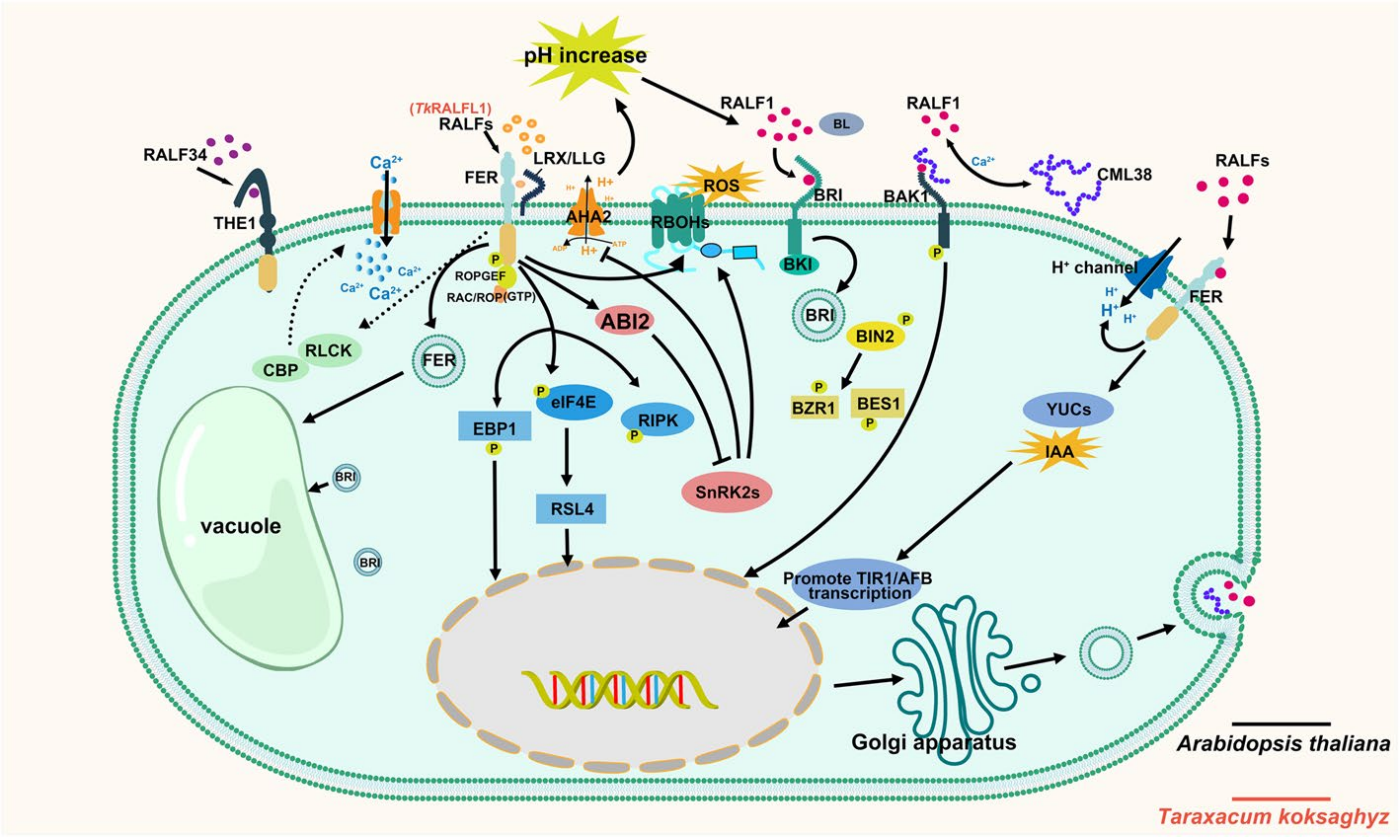
RALFs act as endogenous signaling peptides to regulate root development by controlling plant cell expansion. RALFs phosphorylate AHA2 on the plasma membrane through receptor complex, inhibiting proton transport, thereby inducing alkalinization of the extracellular matrix (Haruta, et al. 2014). RALFs–FER promotes the expression of auxin synthesis genes YUCs, thereby inducing TIR1/AFB transcription and persistently inhibiting root elongation (Li, et al. 2022). Increased pH promotes the dissociation of BRI and BAK1, allowing AtRALF1 and CML38 to bind to BAK1 (Campos, et al. 2018; Dressano, et al. 2017), inhibiting BL-induced cell elongation. RALFs activate FER by increasing the phosphorylation level of FER. The FER–RopGEF–ROP/RAC complex interacts with ABI2, phosphorylates and activates ABI2, and negatively regulates ABA signaling pathways (Chen, et al. 2016; Yu, et al. 2012). Activated FER mediates signal transduction from the cell membrane surface to the nucleus by phosphorylating EBP1, eIF4E, and RIPK. EBP1 appears to repress the transcription of CML38 (Li, et al. 2018; Liu, et al. 2011; Zhu, et al. 2020). RALF1 mediates endocytosis of FER and BRI into the vacuole (Yu, et al. 2020). In root cells, RALFs–FER probably triggers the increasing of cytoplasmic Ca^2+^ concentration in response to receptor-like cytoplasmic kinase (RLCK) and Ca^2+^-binding protein (CBP) (Fuglsang, et al. 2007). Annotated Arabidopsis RALF homologs in other species

Members of the *Cr*RLK1L family are involved in regulating cell expansion (Guo, et al. 2009a, 2009b), and RALF peptides regulate root system development through *Cr*RLK1L redundancy (Fig. 3) (Abarca, et al. 2021; Gonneau, et al. 2018; Morato do Canto, et al. 2014; Zhu, et al. 2020).

Root expansion is accompanied by the relaxation of cell wall structure, modification of cell-wall components, and alteration of cell wall metabolic gene activity (Hamann 2015). Acidification of the apoplast has been shown to play a crucial role in activating cell wall-loosening enzymes and stimulating root cell expansion (Barbez, et al. 2017; Wu, et al. 2007). The RALF1–FER module induces alkalinization of the extracellular matrix by phosphorylating H^+^-ATPase AHA2 at the plasma membrane and inhibiting proton transport (Haruta, et al. 2014). The increase in pH of the external environment is believed to be the leading cause of the inhibition of cell elongation (Barbez, et al. 2017; Li, et al. 2021). In *Nicotiana attenuata*, the maintenance of the short root hair phenotype of the mutant *irRALF* with inverted silencing of *Na*RALF exhibits a high pH dependence, implying that RALF controls root hair development by maintaining appropriate pH levels (Wu, et al. 2007). Li, et al. (2022) have raised conflicting views that RALFs induce transient proton influx through FER, which triggers apoplast alkalization and triggers root growth inhibition. They also reported H^+^-ATPase is not involved in RALF1-induced growth inhibition, as the RALFs–FER maintains long-term root growth inhibition via the TRANSPORT INHIBITOR RESPONSE 1/AUXIN SIGNALING F-BOX PROTEIN-mediated auxin biosynthetic pathway.

Studies have found that RALF appears to be involved in BRASSINOSTEROID (BR)-mediated root morphogenesis (Bergonci, et al. 2014a; Bergonci, et al. 2014b). RALF, as a negative regulator of cell elongation, exhibits a mutually antagonistic relationship with BR (Bergonci, et al. 2014a; Bergonci, et al. 2014b; Srivastava, et al. 2009). The protein BAK1 likely acts as part of the receptor complex that senses RALF1 and regulates cell expansion (Dressano, et al. 2017). Previously, alkalinization of the extracellular matrix was often seen as a prerequisite for inhibiting cell expansion (Haruta, et al. 2014). RALF1 mediates the inhibition of root cell elongation by inducing the phosphorylation of BAK1 but is not dependent on BAK1 for cytoplasmic Ca^2+^ accumulation and extracellular alkalinization (Dressano, et al. 2017). The binding of BAK1 to *At*RALF1 appears to be downstream of extracellular matrix alkalinization and Ca^2+^ turbulence as a way to antagonize BR signaling. Preferential binding of brassinolide (BL) to BRI at the plasma membrane requires an acidic pH requirement of the apoplast (pH 4–5.7), and pH changing drives dissociation of the complex. It is speculated that *At*RALF1-induced alkalinization of the extracellular matrix can dissociate the acidic environment-dependent binding of the BRI–BL–BAK1 complex (Belkhadir and Jaillais 2015; Dressano, et al. 2017). RALF1–FER interaction promotes BRI1 endocytosis, demonstrating that RALF accelerates the dissociation of the BL-sensing complex (Yu, et al. 2020), implying that RALF and BR likely share certain signaling pathway members and compete with each other. However, the dose-dependent effect of BR on root elongation may deepen the complexity of its interaction with RALF signaling (Clouse 2011; Zhu, et al. 2013).

In addition to cell expansion caused by cell wall loosening, the size of the vesicle, the largest organelle in the plant cell, also affects root cell expansion. The LRX3/4/5-FER mediating kinase signaling is critical for the cell wall sensing mechanism and controls intracellular vesicle expansion (Dunser, et al. 2019). RALF1-induced root length inhibition in *lrx3/4/5* mutants is relatively more intense than in the *fer* mutant (Dunser, et al. 2019), suggesting an incomplete overlap of LRX–FER triggered cell wall sensing with RALF1-FER activated signaling. However, the interaction between LRX3/4/5 and RALFs also suggests a possible unknown role for RALF in vesicle expansion (Zhao, et al. 2018). In addition to inhibiting root cell expansion, RALF may also regulate root gravitropism. The receptor FER is involved in auxin-regulated transient alkalization of the apoplast, triggering root gravitropism through asymmetric alkalinization of roots (Barbez, et al. 2017). Studies have found that RALF–FER can induce auxin biosynthesis and activate auxin signal transduction (Li, et al. 2022). It is speculated that RALF as a ligand for FER is most likely involved in regulating the groundward growth of roots.

RALF-mediated regulation of the root system may involve Ca^2+^ signaling and potentially other signals (Haruta, et al. 2014). Ca^2+^-sustained exocytosis is required for root cell expansion (Demidchik and Maathuis 2007). RALF1 was reported to act as an endogenous peptide inducing increased cytoplasmic Ca^2+^ concentrations (Haruta, et al. 2008). Recently, the Ca^2+^-permeable channel protein MLO was identified in pollen tubes functioning downstream of RALF, maintaining the Ca^2+^ gradient and mechanical strength in pollen tubes (Gao, et al. 2023). However, Ca^2+^ channels associated with the RALF–*Cr*RLK1L pathway participating in the regulation of the root system have not yet been identified. Researchers have found that calmodulin-like protein 38 (CML38), a sensor for sensing Ca^2+^ oscillations, binds to *At*RALF1 in a Ca^2+^ and pH-dependent manner extracellularly, reflecting that CML38 is necessary for *At*RALF1 to mediate the short root phenotype (Campos, et al. 2018). However, as CML38 is a secreted peptide, it does not appear to be the cause of the elevated intracellular Ca^2+^ concentration. We can only speculate on how RALF functions as a ligand for calcium mobilization. The blocking of the Ca^2+^ channel inhibited the alkalization of the extracellular matrix after RALF treatment (Gjetting, et al. 2020), and thus, Ca^2+^-initiated signaling cascades may be a prerequisite for RALF to activate H^+^ transfer and inhibit root cell elongation. The protein PKS5, an upstream receptor kinase of AHA2, can bind the Ca^2+^-binding protein SOS3 and SOS3-like calcium-binding protein 1 (SCaBP1) and trigger an increase in Ca^2+^ concentration (Fuglsang, et al. 2007). In addition, the mechanism underlying the activation of AHA2 in the unique acidic environment seems to imply a start-brake feedback regulation from the mechanism underlying RALF alkalinization (Hoffmann, et al. 2019). The generation of local ROS in the root is necessary to induce the polar growth of root hair (Foreman, et al. 2003). Plants control abscisic acid (ABA) signaling through the FER–ROPGEF–ARAC/ROP module and regulate ROS levels in roots through downstream NADPH oxidase to control the polar growth of root hairs (Chen, et al. 2016; Duan, et al. 2010). RALF upstream of this ROS regulatory pathway and has been shown to interact with ABA to regulate the growth of plant roots (Chen, et al. 2016).

### Typical RALFs function as intercellular and intracellular stimuli signals

**Fig. 4:**
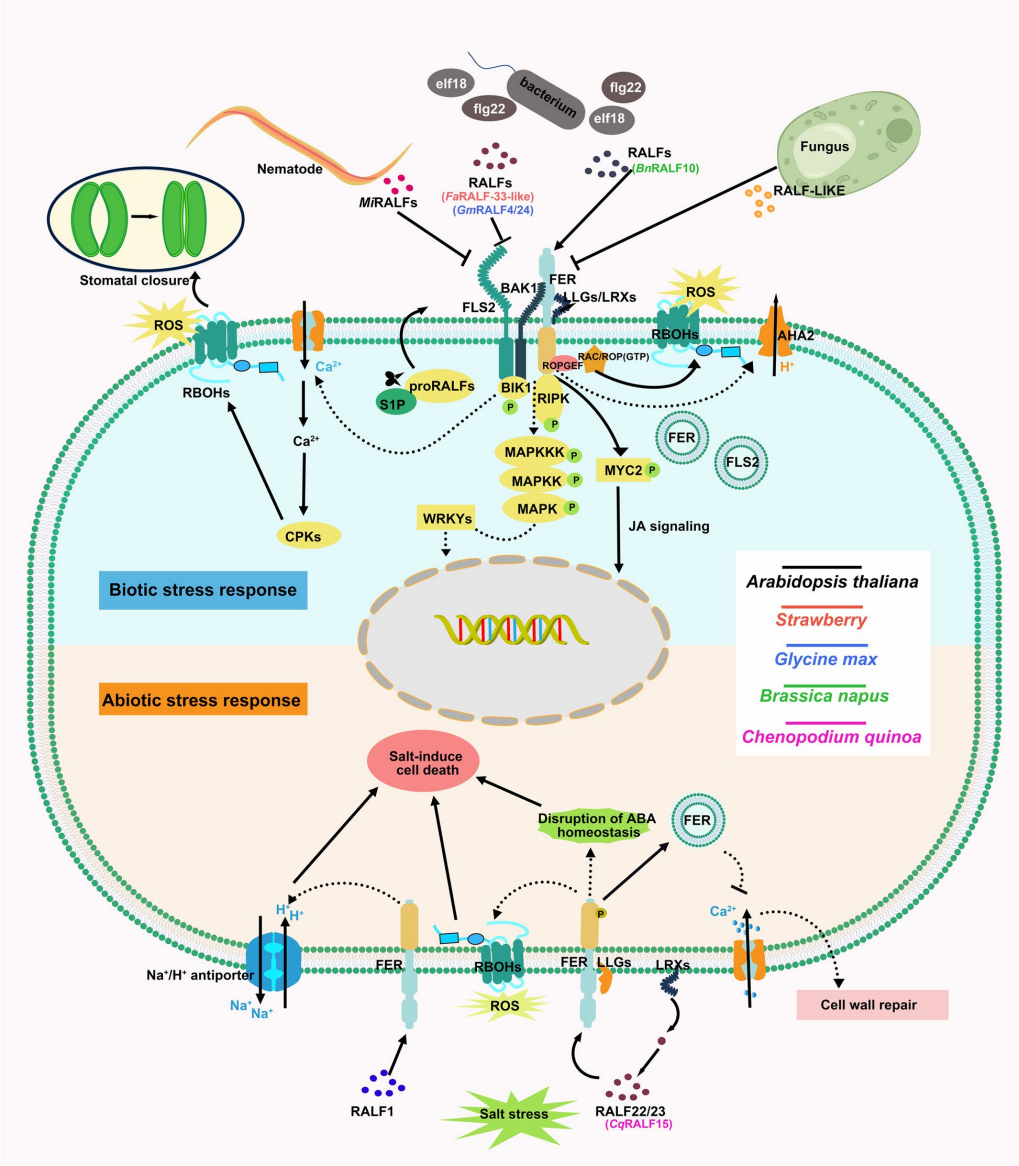
RALFs exhibit different responses in the face of external stimuli. The upper side of the cell shows the activity of RALFs under biotic stress: FER acts as a scaffold to mediate the immune complex formation and sense the stimulation of RALFs. RALFs respond to the stimulation of pathogenic microorganisms, and show positive or negative regulation of plant immunity (Stegmann, et al. 2017a). The RALF-receptor complex induces phosphorylation of downstream BIK1 and participates in MYC2-regulated jasmonic acid signaling (Guo, et al. 2018; Shen, et al. 2017). RALF is likely to regulate plant immunity by activating or inhibiting immune-related responses such as Ca^2+^ oscillations, MAPK cascades, and ROS bursts. The influx of Ca^2+^ regulates ROS production during immune signaling and controls stomatal responses to pathogenic microorganisms through the activation of calcium-dependent protein kinases (CPKs) (Li, et al. 2014). A range of fungi and nematodes can also secrete plant RALF homologs to suppress host immune responses and increase host disease susceptibility (Duan, et al. 2022). The lower side of the cell shows the activity of RALFs under abiotic stress: under salt stress, the extracellular domain of FER and co-receptor LLGs sense the cell wall perturbation caused by Na^+^, trigger intracellular Ca^2+^ transients, and initiate cell wall repair (Feng, et al. 2018). Increased Na^+^ induces perturbation of the cell wall, dissociation of RALF22/23 from LRXs, and promotes RALF22/23-induced internalization of FER, and finally RALF inhibits the signal transduction ability of FER (Zhao, et al. 2018). The RALF–LRX–FER module likely induces cell death under salt stress through loss of ABA homeostasis, accumulation of ROS, and increased ion concentration (Zhao, et al. 2021). Annotated Arabidopsis RALF homologs in other species

Mobile peptides are necessary for cell-to-cell communication and cell-to-external environment communication in plants (Endo, et al. 2013; Tabata, et al. 2014; Takahashi, et al. 2018). RALF–*Cr*RLK1L module specifically enables a signaling pathway to adapt to environmental changes through intercellular and intracellular signal communication (Fig. 4) (Table 1, Subfamily-II, IV).

#### RALFs percept the intercellular pathogenic stimuli signals

Leucine-rich pattern recognition receptors have high binding efficiency for peptide elicitors. A series of LRKs have been identified as receptors of pathogen-induced peptides involved in the immune response (Huffaker, et al. 2006; Igarashi, et al. 2012; Tang, et al. 2015; Wang, et al. 2021; Yamaguchi, et al. 2010, 2006). Among these receptors, FER acts as a scaffold to mediate the formation of a recognition complex involving EF-TU RECEPTOR (EFR)/ FLAGELLIN-SENSING 2 (FLS2) and BAK1 on the membrane surface. RALFs act as ligands to activate this receptor complex and trigger downstream immune responses (Stegmann, et al. 2017b).

RALFs show two completely different behaviors in response to stimulation by pathogenic microorganisms. RALFs that can be cleaved by *At*S1P, such as RALF23/33, dependent receptor kinase FER, can inhibit the generation of ROS induced by elf18 and negatively regulates plant immunity. RALFs lacking the S1P cleavage site, such as RALF17, promote ROS bursts (Stegmann, et al. 2017b). The immune response induced by RALF may regulate plant immunity by integrating signaling pathways, inhibiting or sharing a series of downstream immune-related reactions, including induction of ROS outbreaks, activation of the mitogen-activated protein kinase (MAPK) cascade, hormone level fluctuations, and Ca^2+^ influx (Couto and Zipfel 2016; Guo, et al. 2018; Kadota, et al. 2014; Li, et al. 2014). Recently, FER and LRX were found to rapidly perceive the stimulation of RALF23 and further regulate the nanoscale organization of FLS2 and BAK in the plasma membrane, which provided a new understanding of the suppression of immune signaling transmission by RALF23 (Gronnier, et al. 2022).

Guo, et al. (2018) further explained the mechanism underlying the negative regulation of plant immunity by the RALF23-FER module. The receptor FER phosphorylates and destabilizes the transcription factor MYC2 to increase salicylic acid accumulation, which, in turn, positively regulates plant immunity (Guo, et al. 2018; Zheng, et al. 2012). RALF23 is involved in MYC2-regulated jasmonic acid signaling dependent on FER. Overexpression of RALF23 can increase the accumulation of MYC2, thereby aggravating susceptibility to pathogen-mediated disease in the host (Guo, et al. 2018).

RPM1-induced protein kinase (RIPK) and glycine-rich RNA binding protein 7 (GRP7), which have been identified as downstream of RALF–FER, were also found to be involved in plant immune regulation as immune effector genes in addition to their involvement in regulating root responses (Du, et al. 2016; Liu, et al. 2011; Nicaise, et al. 2013; Wang, et al. 2020b). However, the immune signaling events induced by RALF through RIPK or GRP7 remain unclear.

In addition, RALF is involved in fungal invasion-induced signal transduction events (Duan, et al. 2022). *Gm*RALF4 and *Gm*RALF24, which are highly homologous to *At*RALF23, are involved in the immune response to *F. oxysporum*, and soybean hypocotyls treated with *Gm*RALF4 and *Gm*RALF24 were less resistant to *F. oxysporum* (Liu, et al. 2022b). In *Brassica napus* plants, *Bn*RALF10 elicits immunity to *Sclerotinia sclerotiorum* by stimulating ROS generation through FER (He, et al. 2022).

RALF homologs have been identified in 26 species of plant pathogenic fungi and several bacteria (Thynne, et al. 2017). We are not clear on the evolutionary origin of RALFs in pathogens. Researchers have raised possible conjectures that RALFs in pathogens may have been acquired via horizontal gene transfer from plants or may have evolved convergently with RALFs in plants (Masachis, et al. 2016; Thynne, et al. 2017; Wood, et al. 2020). The alkalinization of the extracellular matrix induced by* F. oxysporum* RALF homolog reduced the immune response to *F. oxysporum* by activating the phosphorylation of immune-associated MAPK cascade reactions (Masachis, et al. 2016). It's not the only case wherein fungi secrete plant RALF analogs to increase their infestation virulence by posing the identity of plants (Merino, et al. 2019; Thynne, et al. 2017; Wood, et al. 2020).

RALFs also bring the opportunity for parasitism to root-knot nematodes. Root-knot nematodes *Meloidogyne incognita* secrete *Mi*RALF1 and *Mi*RALF3, which mimic plant RALF peptides interacting with FER in the host plant to suppress host immune responses and enhance parasitic virulence (Zhang, et al. 2020a). The second disulfide bond of *At*RALF1 is necessary for inhibiting flg22-induced ROS generation. In the nematode peptide RALF-like, the functional second disulfide bond is also retained (Zhang, et al. 2020a). The invasion of RALF-like peptides encoded by nematodes into soybean showed a similar virulence mechanism (Zhang, et al. 2021a).

The role of peptides in immunity is twofold, specific peptides can amplify immune signals by forming positive feedback loops and promote local and systemic immune defenses (Huffaker, et al. 2006; Stegmann, et al. 2017b; Wang, et al. 2021; Yamaguchi, et al. 2010). In contrast, another series of plant peptides negatively regulate plant immunity, allowing plants to adjust rapidly and flexibly in the face of environmental changes (Igarashi, et al. 2012; Stegmann, et al. 2017b). The dual effects of RALFs on immune regulation may be a powerful adaptive mechanism developed by plants to adapt to rapidly changing environments. In cases where the threat posed by external pathogens is insufficient to affect plant survival, a rapid resource allocation adjustment can make developmental growth superior to the immune response of the plant (Segonzac and Monaghan 2019).

#### RALFs mediate the signaling transduction of abiotic stress stimulation

The receptor FER acts as a direct sensor for sensing cell wall perturbations and transmits external stimuli into the cell. However, RALFs negatively regulate response to salt stress by blocking FER signaling (Feng, et al. 2018; Lin, et al. 2022; Zhao, et al. 2021; Zhao, et al. 2018). Under salt stress conditions, the extracellular structural domain of FER inhibits salt stress-induced cell wall loosening by binding to pectin (Feng, et al. 2018). The down-regulation of genes implicated in cell wall modification in *lrx3/4/5* mutants after salt stress treatment similarly suggests that LRX3/4/5 may be involved in maintaining the integrity of the cell wall under salt stress (Zhao, et al. 2021). The LRX3/4/5–*At*RALF22/23–FER module links salt stress-induced cell wall perturbations to internal signaling. A high concentration of Na^+^ extracellularly induces the dissociation of RALF22/23 from LRX3/4/5, thereby facilitating RALF22/23-induced internalization of FER. RALF22/23 deprived the signal transmission capacity of FER, thereby negatively regulating the salt tolerance function of FER (Zhao, et al. 2018). Subsequently, Zhao, et al. (2021) proposed LRX3/4/5–*At*RALF22/23–FER module-mediated salt stress-induced plant death depends on ABA accumulation and upregulation of ROS generation.

Arabidopsis G-protein *β* subunit (AGB1) as a heterotrimeric G-protein β subunit regulates stomatal movement in *A. thaliana* (Temple and Jones 2007; Yu and Assmann 2015). The *agb1* mutant showed increased Na^+^ accumulation under salt stress conditions, likely due to the higher stomatal conductance in these mutants than in the wild type resulting in stronger transpiration, triggering increased Na^+^ translocation from the root to the shoot. The increased Na^+^ translocation, in turn, triggers a hypersensitive response of *agb1* to salt stress due to ionic stress (Yu and Assmann 2015). Subsequent studies revealed that RALF1 was involved in AGB1-mediated regulation of stomatal conductance through the receptor FER, and the RALF1-mediated stomatal response was likely dependent on FER kinase activity. RALF1 responds to the stimulus by promoting stomatal closure and inhibiting stomatal opening (Chakravorty, et al. 2018; Yu, et al. 2018).

Is RALF1 involved in the salt stress response in plants by regulating stomatal conductance with AGB1 and FER? The proteins AGB1 and FER showed synergistic effects in response to salt stress, but RALF1 caused salt toxicity in plants by increasing ion concentrations, independent of AGB1, and did not induce ROS accumulation. The *fer* mutant showed no Na^+^ accumulation post-NaCl treatment, implying that RALF1-FER is involved in a cell death mechanism underlying salt stress independent of AGB1 (Yu and Assmann 2018).

Like Arabidopsis, crops also have multiple RALF-resistant genes, providing potential genetic resources for us to breed multi-resistant crops. RALFL33 in cotton is likely to be a key gene regulating cold and salt stress. Through comparative transcriptome analysis and gene evolution analysis on four kinds of diploid D-genome cotton, Xu, et al. (2020) discovered a potential regulatory network centered on RALFL33. In moss *Physcomitrium patens*, *Pp*RALF3 is not involved in promoting protonema tip growth and elongation (Ginanjar, et al. 2022). However, knockout lines of *PpRALF2* and *PpRALF3* show increased resistance to bacterial and various fungal pathogens. The *PpRALF3* is more resistant to paraquat and NaCl, and has longer protonemal segments after stress treatment (Mamaeva, et al. 2023), showing excellent agricultural application traits. *Chenopodium quinoa* is a halophyte suitable for planting in infertile soils. *Cq*RALF–FER module is conservatively involved in the regulation of salt tolerance in *Chenopodium quinoa*, which may largely address the problem of land salinization (Jiang, et al. 2022).

### Information gap on atypical RALFs lacking conserved motifs needs to be filled

Most RALFs with YIXY motifs, S1P cleavage sites, and stable disulfide bonds functionally inhibit cell expansion and cause alkalinization of the extracellular matrix, therefore, are involved in the regulation of plant development and the response to external stimuli. In addition, atypical RALFs lacking several conserved motifs were found to exert physiological effects consistent or inconsistent with typical RALF (Table 1, Subfamily-I, III). Compared with the depth research on typical RALF, the research on atypical RALF still has a long way to go. Atypical RALFs occur in a large proportion in crops (Fig. 1), and these potential genetic resources need to be explored in the future.

Non-interchangeability of the extracellular domains of *Cr*RLK1L members suggests ligand binding specificity during plant reproduction (Kessler, et al. 2015). Moreover, except for the typical RALFs implicated in reproductive development (Gao, et al. 2023; Ge, et al. 2017; Ge, et al. 2019; Mecchia, et al. 2017; Xiao, et al. 2019), other atypical RALFs expressed in reproductive tissues can serve as specificity ligands for unknown receptors. These atypical RALFs may be irreplaceable in plant reproduction and development (Gao, et al. 2022; Ge, et al. 2017). Thus far, only RALF6, 7, 16, 36, and 37 lacking S1P sites and expressed in pollen and pollen tubes have been found to establish mobilizable polyspermy blocks by binding to the FER/ANJ/HERK1 receptor complex, to prevent polyspermy and maintain double fertilization. RALFs play a molecular gating role, sustaining the one-to-one pattern of pollen tubes and ovules and adjusting their strategy to allow secondary pollen tubes to release polyspermy blocks when fertilization fails, improving reproductive efficiency (Zhong, et al. 2022).

RALFs with S1P sites can negatively regulate plant immunity, and conversely, RALFs lacking S1P sites, represented by RALF17, can promote the elf18-induced generation of ROS by activating FER (Stegmann, et al. 2017b). However, applying 34 kinds of RALF peptides in vitro revealed that the role of RALF in immunomodulation does not only rely on S1P sites to split the function (Abarca, et al. 2021).

Certain other atypical RALF functions are constantly being explored. Overexpression of *At*RALF8, which also lacks the S1P site, was found to have enhanced sensitivity to drought and nematode infection (Atkinson, et al. 2013). It is implied that *At*RALF8 does not appear to improve tolerance to drought and nematode infestation by inducing elevated ROS generation (Abarca, et al. 2021). RALF8 can also initiate downstream signals for root length inhibition via receptor FER (Frederick, et al. 2019). The receptor FER is critical for most RALF peptides to sense ligand and transduction signals (Abarca, et al. 2021). RALF36 is relatively more potent than RALF33 in inhibiting root length. Increased Ca^2+^ concentrations and pH in *fer* mutants treated with RALF36 predicted the presence of other receptor kinases that play a dominant role downstream of RALF36 (Gjetting, et al. 2020). The same RALF peptide may have multiple roles in specific tissues and cells through different or polymeric receptors (Abarca, et al. 2021). The complex redundancy and specificity of peptide signaling functions present significant difficulties for our research.

The research on atypical RALF is limited as the function of atypical RALF, which does not have any conserved motifs, is currently unknown. Campbell and Turner (2017) found that RALFs without conserved motifs accounted for one-third of the total RALFs, which were evolutionarily isolated from typical RALFs and had gene expression patterns and physicochemical properties significantly different from typical RALFs. Thus, they could not be studied as typical RALFs. Therefore, atypical RALF may have critical functions that we have not yet identified in the development regulation of plants, and we may need to analyze new receptors and explore them from a new perspective.

## Application potential of RALFs in agriculture

**Fig. 5:**
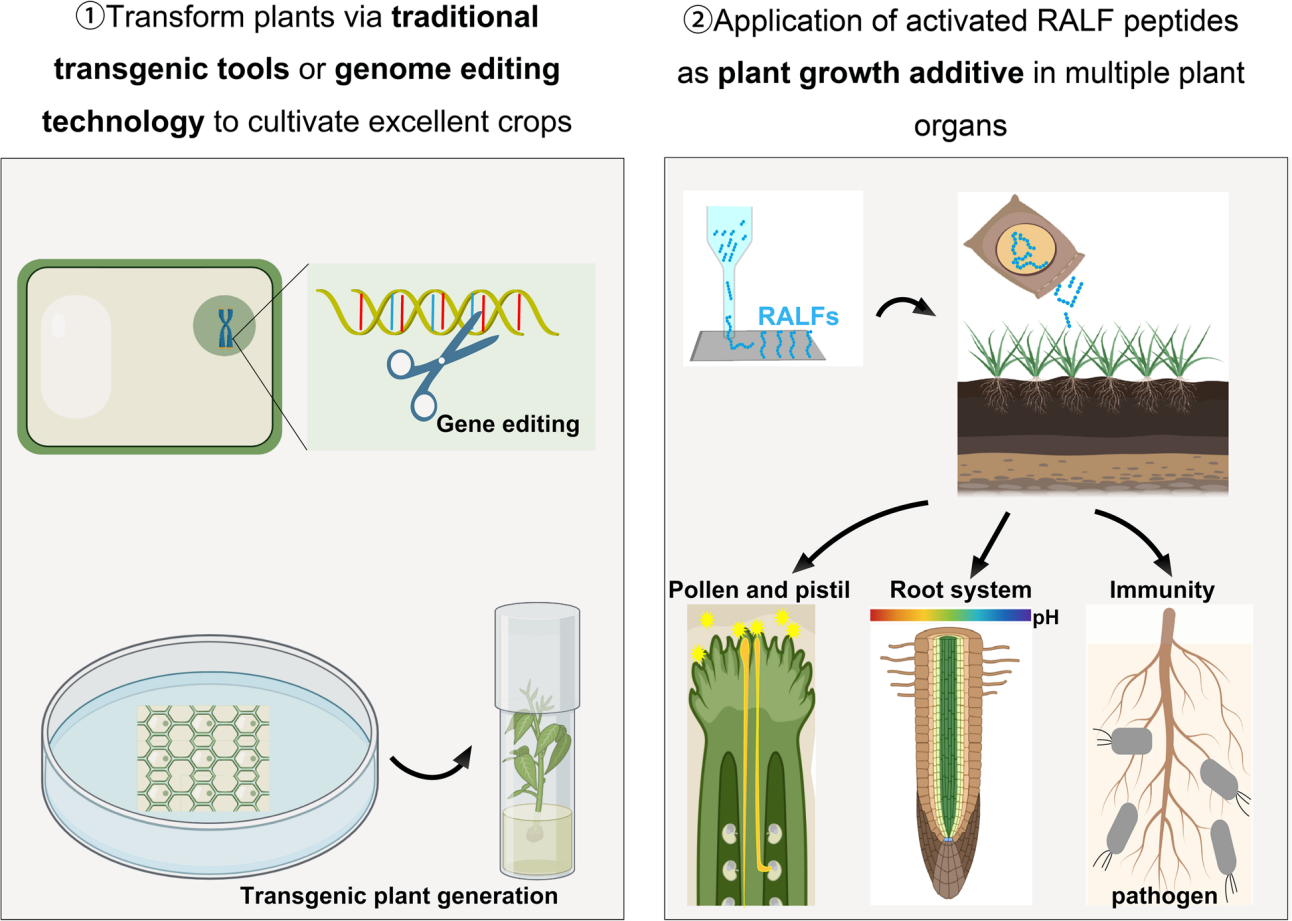
Application potential of RALFs in agriculture. Traditional transgenic tools or gene editing techniques were used to create transgenic plants of RALFs, for relieving the growth inhibition caused by RALFs and amplifying the positive regulatory role of RALFs. We can also develop plant growth additives related to RALFs: (1) applying RALFs to stigma to improve plant reproduction, (2) applying RALFs to root system to improve the adaptation to acidic culture substrates and cultivate crops with excellent root traits, and (3) applying antimicrobial RALFs to plants to increase the resistance of crops

To fully utilize the regulatory role of RALF and convert it into productivity, we propose the following two strategies (Fig. 5): (1) using traditional transgenic tools or genome editing technology to relieve growth inhibition caused by RALF peptides and amplify the positive regulatory role of RALF, and (2) using information related to RALF structure to develop growth additives for regulating plant growth and improving crop yield through exogenous applications.

### Application of RALFs in improving plant reproduction

RALF plays a molecular gating role in precisely regulating plant reproduction at the pollen–stigma interface, the pollen tube extension process, and the pollen tube–synergid cell interface (Cheung, et al. 2022). At the pollen–stigma surface, it functions with different kinds of polypeptides to allow precise female–male communication (Liu, et al. 2021). It ensures the smooth transport of sperm cells and other contents during the pollen tube extension process (Ge, et al. 2017; Zhou, et al. 2021). At the pollen tube–synergid cell interface, it facilitates the release of sperm cells and establishes a mobilizable polytubey mechanism, which provides the prerequisite for adequate male and female gamete binding (Gao, et al. 2022; Zhong, et al. 2022).

RALFs act as triggers for the barrier at the transmitting tract septum, preventing multiple pollen tubes from penetrating this barrier and ensuring a relationship of one pollen tube corresponding to one ovule. However, in case of fertilization failure, RALFs allow secondary pollen tubes to undergo fertilization recovery (Zhong, et al. 2022). Compared with female reproductive organs, changes in temperature and humidity significantly affect pollen activity (Dupuis and Dumas 1990; Jiang, et al. 2019; Jin, et al. 2013). Therefore, in unfavorable environments, pollen content is relatively more likely to be strictly limited, and RALF-mediated regulation of the polytubey block mechanism in multi-species plants seems to prolong the chance of fertilization and improve crop fertility (Kasahara, et al. 2013; Zhong, et al. 2022). The improvement of seed fertility by RALF at the molecular level led us to consider whether the application of RALF could be designed effectively to promote the probability of fertilization in flowering plants, thereby increasing the yield potential of crops (Liu, et al. 2021; Zhong, et al. 2022). However, RALFs have not adequately shown their application in agriculture yet, and in-depth research on improving the application value of RALF in practical agricultural production is needed.

### Application of RALFs in the regulation of root development and adaptation to acidic substrates

The gradual acidification of arable land has become one of the major problems limiting modern agriculture. In acidic soil environments with pH below 5.0, Al^3+^ formation triggers considerable plant damage (Szurman-Zubrzycka, et al. 2021). The toxic effects of Al^3+^ can be mitigated effectively by increasing plant root pH in agricultural applications. In wheat plants, loss of root tip H^+^-ATPase activity raises root pH and thus reduces the toxic effects of Al^3+^ (Hayashi, et al. 2005; Yang, et al. 2011). We proposed to envisage whether RALF could neutralize Al^3+^ toxicity in acidic environments. Boron alleviates the inhibition of aluminum toxicity on growth in a pH-dependent manner and the application of RALF to increase root pH could partially replace the application of boron in reducing Al^3+^ toxicity, thus improving the fertility and sustainability of acidic culture substrates (Yang, et al. 2022).

A wide variety of RALFs inhibit root growth, therefore, we can use traditional transgenic tools or CRISPR/Cas9 system to mutate functionally activated RALFs to breed crops with high biomass in root traits, more suitable for cultivation with more economic benefits. For example, the roots of *Taraxacum koksaghyz*, a potentially important source of inulin and natural rubber, were harvested after knockout of *Tk*RALFL1. The *tkralfl1* mutant has higher primary root biomass than in the wild type while allowing for higher planting densities, effectively increasing the economic value of *tkralfl1* mutant plants (Wieghaus, et al. 2019). The *Hb*RALF–*Hb*FER module is also involved in rubber biosynthesis by regulating the pH of rubber latex (Sui, et al. 2023).

### Antimicrobial application of RALFs in crops

RALF, a plant immunomodulator, is involved in host–pathogen crosstalk (Duan, et al. 2022; Masachis, et al. 2016; Song, et al. 2021; Zhang, et al. 2020a; Zhang, et al. 2021a), and also plays a crucial role in the immune response of crops. *Os*RALFL7 (LOC_Os01g25560) and *Os*RALFL8 (LOC_Os02g44940) were able to be induced by the fungus *Magnaporthe oryzae* (Wang, et al. 2020c). The study reported that inoculation of *Fg*RALF (*At*RALF1 homolog) heterologously expressed using the BSMV vector in wheat spikelet slightly promoted *Fusarium graminearum* colonization. It shows that the RALF homolog in *F. graminearum* exerts part of the infective virulence on the host during gibberellic disease infection (Wood, et al. 2020). The role of RALF in promoting pathogen colonization in Arabidopsis, oilseed rape, and soybean, similarly demonstrates that specific RALFs decrease host immunity in response to fungal and nematode infections (He, et al. 2022; Liu, et al. 2022b; Masachis, et al. 2016; Zhang, et al. 2020a; Zhang, et al. Zhang, et al. 2021a). This information provides us with a way to develop resistant crops by artificially mutating or silencing the RALF susceptibility genes to limit the ability of pathogens to cause disease in the host. For example, the silencing of *Fa*RALF-33-like in ripening strawberries suppressed fungal colonization in fruit, effectively reducing economic losses caused by anthracnose disease (Merino, et al. 2019).

Utilizing the regulatory role of RALF, we can improve plant resistance to external stress, artificially regulating the abundance of beneficial bacteria. *Pseudomonas fluorescens* is a biological control strain that can reduce plant disease. Plants control ROS generation through the RALF23–FER module, thereby controlling the level of *P. fluorescens* independent of the jasmonic acid signaling pathway and the immune scaffolding function of FER (Song, et al. 2021). Exogenous application of RALF23 improves the enrichment of *P. fluorescens* under specific requirements.

Plant-expressed polypeptides or polypeptide reagents produced by industrial mass fermentation using microorganisms have been proven to exert apparent antibacterial effects (De Samblanx, et al. 1996; Hoelscher, et al. 2022; López-García, et al. 2000; Shanmugaraj, et al. 2021). A series of RALFs lacking the S1P cleavage site are against specific pathogenic bacteria and trigger plant defense responses through exogenous application (Abarca, et al. 2021; Stegmann, et al. 2017b). Therefore, exploring the function of RALFs provides novel options for using antimicrobial peptides to increase disease resistance in crops.

## Summary and prospect

In the two decades since RALF was discovered, numerous studies have made remarkable progress in elucidating the role of RALF in plant development and response to external stimuli. The *Cr*RLK1Ls and co-receptors sense different RALF, and independently play an uncoupled role in regulating plant development through signaling cascades (Mang, et al. 2017; Zhu, et al. 2021). Experiments involving the exchange of extracellular structural domains imply that ligand-receptor-specific recognition is fundamental in determining the downstream signaling steps (Kessler, et al. 2015; Liu, et al. 2022a). Compared with the study of RALF in Arabidopsis, research on RALFs in crops is still limited, and an in-depth study on improving the application value of RALF in practical agricultural production is needed. Advances in gene editing technology in recent years allow us to multiple edit RALF peptides with redundant functions (Chen, et al. 2022; Zhang, et al. 2021b), efficiently releasing the growth inhibition caused by RALF peptides and increasing crop the biomass. In addition, using RALF as a green plant hormone has important practical significance in improving agricultural traits. Realizing the industrial production of activated RALF peptides and the large-scale use of RALFs as plant growth regulators are what we hope to achieve in the future.

## Supplementary Information

**Additional file 1.** 

## Data Availability

Not applicable.

## Abbreviations

ABA: Abscisic acid
ANJ: ANJEA
ANX: ANXUR
AGB1: Arabidopsis G-protein *β* subunit
BL: Brassinolide
BR: BRASSINOSTEROID
BUPS: BUDDHA'S PAPER SEAL
CBP: Ca^2+^-binding protein
CPKs: Calcium-dependent protein kinases
CML38: Calmodulin-like protein 38
*Cr*RLK1Ls: Catharanthus roseus Receptor-like kinase 1-like proteins
EFR: EF-TU RECEPTOR
FER: FERONIA
FLS2: FLAGELLIN-SENSING 2
GRP7: Glycine-rich RNA binding protein 7
GPI-AP: Glycolphosphatidylinositol-anchored protein
HERK1: HERCULES RECEPTOR KINASE 1
LRKs: Leucine repeat receptor kinases
LRXs: Leucine-rich repeat extension proteins
LRE: LORELEI
LLG3: LORELEI-like glycolphosphatidylinositol-anchored protein 3
MLO: MILDEW RESISTANCE LOCUS O
MAPK: Mitogen-activated protein kinase
NO: Nitric oxide
NTA: NORTIA
PCP-B: POLLEN COAT PROTEIN B
RALF: Rapid alkalinization factor
ROS: Reactive oxygen species
RLCK: Receptor-like cytoplasmic kinase
RBOHs: Respiratory burst oxidase homologs
RIPK: RPM1-induced protein kinase
S1P: Site-1 protease
SCaBP1: SOS3 and SOS3-like calcium-binding protein 1
