# Supplementary material for: Rapid alkalinization factor: function, regulation, and potential applications in agriculture
Source: Stress Biol. 2023 May 29;3(1):16. doi: 10.1007/s44154-023-00093-2 (PMC10442051; doi:10.1007/s44154-023-00093-2)
Supplement: Supplementary file 1 — Additional file 1. [file 44154_2023_93_MOESM1_ESM.docx]

**Table S1 List of RALF genes identified in five cereals and Arabidopsis.**

| **Gene ID** | **Gene Location** | **Genomic (bp)** | **CDS (bp)** | **Protein (AA)** | **MV (Da)** | **PI** | **Signal Peptide Prediction Probability and Position** |
| --- | --- | --- | --- | --- | --- | --- | --- |
| **Zm00001d029122** | **1:‌59021967..59022404(-)** | **438** | **435** | **145** | **15036.78** | **4.63** | **Pos. 25 and 26. Probability 0.942960** |
| **Zm00001d023856** | **10:‌25214019..25214375(+)** | **357** | **354** | **118** | **12608.18** | **8.86** | **Pos. 20 and 21. Probability 0.971773** |
| **Zm00001d008881** | **8:‌23662010..23662438(-)** | **429** | **426** | **142** | **14480.49** | **9.32** | **Pos. 29 and 30. Probability 0.548649** |
| **Zm00001d041685** | **3:‌133286614..133286988(-)** | **375** | **372** | **124** | **13041.74** | **9.3** | **Pos. 20 and 21Probability 0.974319** |
| **Zm00001d011922** | **8:‌164677723..164678097(+)** | **375** | **372** | **124** | **13123.63** | **8.57** | **Pos.24 and 25. Probability 0.971795** |
| **Zm00001d049233** | **4:‌21990923..21991252(+)** | **330** | **327** | **109** | **12071.95** | **8.35** | **Pos. 31 and 32. Probability 0.963718** |
| **Zm00001d039429** | **3:‌4033017..4033454(-)** | **438** | **435** | **145** | **14826.86** | **9.54** | **Pos. 29 and 30. Probability 0.917162** |
| **Zm00001d040803** | **3:‌66922907..66923248(-)** | **342** | **339** | **113** | **11716.33** | **9.16** | **Pos. 25 and 26. Probability 0.971906** |
| **Zm00001d040297** | **3:‌36319753..36320169(-)** | **417** | **414** | **138** | **13967.76** | **8.61** | **Pos. 27 and 28. Probability 0.968731** |
| **Zm00001d048716** | **4:‌4145643..4146017(-)** | **375** | **372** | **124** | **12534.29** | **8.3** | **Pos. 25 and 26. Probability 0.838366** |
| **Zm00001d009247** | **8:‌47084311..47084655(-)** | **345** | **324** | **108** | **11302.92** | **9.01** | **Pos. 23 and 24. Probability 0.857796** |
| **Zm00001d011921** | **8:‌164602381..164602755(+)** | **375** | **372** | **124** | **12584.26** | **9.49** | **Pos. 35 and 36. Probability 0.945244** |
| **Zm00001d022472** | **7:‌178318703..178319089(-)** | **387** | **384** | **128** | **13163.55** | **6.53** | **Pos. 31 and 32. Probability 0.796655** |
| **Zm00001d036706** | **6:‌98210806..98211135(+)** | **330** | **327** | **109** | **11887.24** | **9.85** | **Not signal peptide** |
| **Zm00001d037738** | **6:‌‌‍135959877..135960266(+)** | **390** | **387** | **129** | **13603.27** | **9.04** | **Pos. 30 and 31. Probability 0.507549** |
| **Brara.H02002** | **A08:‌‌18303232..18303567(-)** | **336** | **333** | **111** | **12705.68** | **9.85** | **Pos. 24 and 25. Probability 0.968967** |
| **Brara.C02008** | **A03:‌‌9991529..9991930(-)** | **402** | **201** | **67** | **6758.87** | **8.35** | **Pos. 27 and 28. Probability 0.977558** |
| **Brara.H00473** | **A08:‌‌5043266..5043961(+)** | **696** | **330** | **110** | **12417.2** | **5.61** | **Pos. 24 and 25. Probability 0.953319** |
| **Brara.G00815** | **A07:‌‌10104647..10104982(-)** | **336** | **333** | **111** | **12658.62** | **9.79** | **Pos. 24 and 25. Probability 0.975204** |
| **Brara.E03362** | **A05:‌‌26998587..26998946(-)** | **360** | **357** | **119** | **12994.78** | **9.35** | **Pos. 22 and 23. Probability 0.981549** |
| **Brara.I01496** | **A09:‌‌9638581..9639121(+)** | **541** | **237** | **79** | **8763.13** | **7.71** | **Pos. 28 and 29. Probability 0.922298** |
| **Brara.H00687** | **A08:‌‌8982448..8983104(-)** | **657** | **345** | **115** | **13336.79** | **6.21** | **Not Signal Peptide** |
| **Brara.A02544** | **A01:‌‌20965619..20966188(+)** | **570** | **225** | **75** | **8397.69** | **9.05** | **Pos. 28 and 29. Probability 0.968553** |
| **Brara.E01029** | **A05:‌‌6060783..6061540(-)** | **758** | **330** | **110** | **12442.38** | **9.75** | **Pos. 20 and 21. Probability 0.920161** |
| **Brara.J00141** | **A10:‌‌770160..770528(-)** | **369** | **366** | **122** | **13343.13** | **8.24** | **Pos. 27 and 28. Probability 0.977827** |
| **Brara.I03505** | **A09:‌ ‌33024938..33025730(-)** | **793** | **282** | **94** | **10374.86** | **7.69** | **Pos. 18 and 19. Probability 0.968487** |
| **Brara.D01995** | **A04:‌ 16737712..16737942(-)** | **231** | **228** | **76** | **8390.68** | **4.96** | **Pos. 26 and 27. Probability 0.977700** |
| **Brara.E03428** | **A05:‌ 27298173..27298433(-)** | **261** | **258** | **86** | **9140.77** | **9.55** | **Pos. 30 and 31. Probability 0.973502** |
| **Brara.J02666** | **A10:‌‌18673502..18673777(-)** | **276** | **273** | **91** | **9728.32** | **9.76** | **Pos. 32 and 33. Probability 0.751346** |
| **Brara.C03615** | **A03:‌‌18590283..18591356(+)** | **1074** | **399** | **133** | **14557.59** | **9.07** | **Pos. 32 and 33. Probability 0.379725** |
| **Brara.E02498** | **A05:‌‌22264439..22264822(-)** | **384** | **381** | **127** | **13897.78** | **9.3** | **Pos. 28 and 29. Probability 0.980808** |
| **Brara.K00416** | **Scaffold18850:‌‌5056..5268(-)** | **213** | **210** | **70** | **7809.53** | **9.83** | **Pos. 22 and 23. Probability 0.977618** |
| **Brara.E03429** | **A05:‌‌27301288..27301539(-)** | **252** | **249** | **83** | **8646.23** | **9.26** | **Pos. 30 and 31. Probability 0.969204** |
| **Brara.G00563** | **A07:‌7179518..7180033(-)** | **516** | **222** | **74** | **7986.4** | **9.06** | **Pos. 22 and 23. Probability 0.978812** |
| **Brara.I00460** | **A09:‌2601462..2602078(-)** | **617** | **348** | **116** | **12650.36** | **9.02** | **Pos. 23 and 24. Probability 0.980180** |
| **Brara.B02569** | **A02:‌18164424..18164732(-)** | **309** | **306** | **102** | **11608.49** | **8.32** | **Pos. 24 and 25. Probability 0.974275** |
| **Brara.D01991** | **A04:‌16708940..16709167(+)** | **228** | **225** | **75** | **8299.45** | **5.6** | **Pos. 26 and 27. Probability 0.642273** |
| **Brara.B02424** | **A02:‌15944666..15944983(-)** | **318** | **315** | **105** | **11870.76** | **9.27** | **Pos. 26 and 27. Probability 0.952592** |
| **Brara.C04456** | **A03:‌23511495..23512991(+)** | **1497** | **501** | **167** | **18703.36** | **9.43** | **Pos. 21 and 22. Probability 0.980441** |
| **Brara.A03261** | **A01:‌27177647..27178179(+)** | **533** | **249** | **83** | **8998.51** | **8.93** | **Pos. 25 and 26. Probability 0.966516** |
| **Brara.I03477** | **A09:‌32820182..32820580(-)** | **399** | **396** | **132** | **15014.9** | **6.28** | **Pos. 26 and 27. Probability 0.977021** |
| **Brara.I00864** | **A09:‌4831330..4831713(-)** | **384** | **381** | **127** | **14370.2** | **7.7** | **Pos. 25 and 26. Probability 0.886620** |
| **Brara.E01107** | **A05:‌6563011..6563241(+)** | **231** | **228** | **76** | **8311.4** | **4.35** | **Pos. 27 and 28. Probability 0.961331** |
| **Brara.A03720** | **A01:‌29778981..29779340(-)** | **360** | **357** | **119** | **13128.83** | **9.03** | **Pos. 22 and 23. Probability 0.981512** |
| **Brara.C03104** | **A03:‌15758135..15758992(+)** | **858** | **354** | **118** | **12910.62** | **9.03** | **Pos. 22 and 23. Probability 0.981909** |
| **Brara.H03105** | **A08:‌23049399..23050054(+)** | **656** | **363** | **121** | **13223.92** | **7.57** | **Pos. 26 and 27. Probability 0.978498** |
| **Brara.B00540** | **A02:‌2473077..2473646(+)** | **570** | **219** | **73** | **8080.44** | **8.75** | **Pos. 28 and 29. Probability 0.972114** |
| **Brara.D00675** | **A04:‌5419196..5419555(+)** | **360** | **357** | **119** | **13516.23** | **8.25** | **Pos. 25 and 26. Probability 0.973398** |
| **Brara.B03385** | **A02:‌28244862..28245360(+)** | **499** | **345** | **115** | **12383.18** | **9.54** | **Pos. 24 and 25. Probability 0.982066** |
| **Brara.C01669** | **A03:‌8091437..8091622(+)** | **186** | **183** | **61** | **6776.21** | **9.34** | **Pos. 29 and 30. Probability 0.974260** |
| **Brara.A02614** | **A01:‌21870935..21871745(-)** | **811** | **453** | **151** | **16717.35** | **9.16** | **Not Signal Peptide** |
| **Brara.C03923** | **A03:‌20387335..20387652(+)** | **318** | **315** | **105** | **11990.95** | **9.41** | **Pos. 23 and 24. Probability 0.974706** |
| **Brara.C01687** | **A03:‌8248148..8248671(+)** | **524** | **225** | **75** | **8378.47** | **4.3** | **Pos. 26 and 27. Probability 0.979634** |
| **Brara.H02223** | **A08:‌19408972..19409280(-)** | **309** | **306** | **102** | **11029.62** | **8.65** | **Pos. 27 and 28. Probability 0.978513** |
| **Brara.G00700** | **A07:‌8950017..8950755(-)** | **739** | **351** | **117** | **13182.24** | **8.78** | **Pos. 22 and 23. Probability 0.976097** |
| **Brara.G01200** | **A07:‌12995572..12995961(-)** | **390** | **387** | **129** | **14407.24** | **7.7** | **Pos. 26 and 27. Probability 0.830206** |
| **Brara.I02958** | **A09:‌28747274..28747609(-)** | **336** | **333** | **111** | **12733.78** | **9.9** | **Pos. 24 and 25. Probability 0.968930** |
| **Brara.A02543** | **A01:‌20959589..20959816(-)** | **228** | **225** | **75** | **8530.8** | **9.38** | **Pos. 28 and 29. Probability 0.970721** |
| **Brara.K01003** | **Scaffold38027:‌2889..3200(-)** | **312** | **309** | **103** | **11620.63** | **9.44** | **Pos. 24 and 25. Probability 0.972455** |
| **Traes_4DL_00F40BD38.1** | **4D:‌291765412..291765807(+)** | **396** | **1188** | **131** | **14173.9** | **5.1** | **Pos. 22 and 23. Probability 0.650827** |
| **Traes_2BL_CD459994C1.1** | **2B:‌779040576..779040923(-)** | **348** | **1044** | **115** | **12271.05** | **5.66** | **Pos. 27 and 28. Probability 0.782356** |
| **Traes_3B_4FAD2EB511.1** | **3B:‌734686335..734686682(-)** | **348** | **1044** | **115** | **11876.33** | **8.27** | **Pos. 23 and 24. Probability 0.973294** |
| **Traes_5DS_C204EBAA9.1** | **5D:‌119253742..119254110(-)** | **369** | **1107** | **122** | **12575.24** | **9.54** | **Pos. 22 and 23. Probability 0.977827** |
| **Traes_1AL_BC5B0BA82.1** | **1A:‌536930603..536931140(-)** | **538** | **1614** | **110** | **11594.32** | **8.86** | **Pos. 33 and 34. Probability 0.976999** |
| **Traes_2AL_1368BE0AD.1** | **2A:‌778160119..778162550(+)** | **2432** | **7296** | **107** | **11385.96** | **6.02** | **Pos. 24 and 25. Probability 0.909115** |
| **Traes_5BS_0AFC3F795.1** | **5B:‌131714674..131715627(-)** | **954** | **2862** | **122** | **12605.27** | **9.54** | **Pos. 22 and 23. Probability 0.977931** |
| **Traes_2DS_F48F4CA0F.1** | **2D:‌63702936..63703652(-)** | **717** | **2151** | **101** | **10581.89** | **6.53** | **Pos. 26 and 27. Probability 0.955489** |
| **Traes_3B_609886503.2** | **3B:‌161374233..161374466(-)** | **234** | **702** | **77** | **7977.1** | **10.04** | **Pos. 28 and 29. Probability 0.429668** |
| **Traes_2AS_37CF96C4E.1** | **2A:‌65395603..65396150(-)** | **548** | **1644** | **100** | **10413.74** | **6.12** | **Pos. 20 and 21. Probability 0.963152** |
| **Traes_1BL_A30620B5E.1** | **1B:‌598925820..598926173(+)** | **354** | **1062** | **117** | **12286.08** | **9.22** | **Pos. 26 and 27. Probability 0.876408** |
| **LOC_Os10g18170** | **Chr10:‌9227677..9228006(-)** | **330** | **327** | **109** | **11234.85** | **8.84** | **Pos. 26 and 27. Probability 0.963600** |
| **LOC_Os04g54090** | **Chr4:‌32234697..32235021(+)** | **325** | **288** | **96** | **10117.06** | **8.89** | **Not Signal peptide** |
| **LOC_Os01g10470** | **Chr1:‌5540629..5541520(-)** | **892** | **438** | **146** | **15138.32** | **8.59** | **Pos. 29 and 30. Probability 0.912360** |
| **LOC_Os01g25540** | **Chr1:‌14480782..14481616(-)** | **835** | **351** | **117** | **11642.07** | **7.64** | **Pos. 25 and 26. Probability 0.967394** |
| **LOC_Os01g70690** | **Chr1:‌40923718..40924053(-)** | **336** | **333** | **111** | **11333.92** | **8.86** | **Pos. 27 and 28. Probability 0.930412** |
| **LOC_Os01g15320** | **Chr1:‌8577199..8577934(-)** | **736** | **393** | **131** | **13154.83** | **8.27** | **Pos. 24 and 25. Probability 0.536496** |
| **LOC_Os01g25560** | **Chr1:‌14486393..14487170(-)** | **778** | **348** | **116** | **11844.41** | **4.89** | **Pos. 28 and 29. Probability 0.965852** |
| **LOC_Os03g22440** | **Chr3:‌12862612..12863022(-)** | **411** | **408** | **136** | **14377.48** | **8.27** | **Pos. 27 and 28. Probability 0.547428** |
| **LOC_Os02g44940** | **Chr2:‌27228829..27229723(+)** | **895** | **357** | **119** | **11811.31** | **9.16** | **Pos. 29 and 30. Probability 0.678210** |
| **LOC_Os12g35670** | **Chr12:‌21691609..21692695(+)** | **1087** | **381** | **127** | **13074.69** | **8.9** | **Pos. 25 and 26. Probability 0.843248** |
| **LOC_Os12g35690** | **Chr12:‌21699858..21700997(+)** | **1140** | **510** | **170** | **17039.32** | **9.55** | **Not Signal peptide** |
| **LOC_Os05g11330** | **Chr5:‌6405567..6406491(-)** | **925** | **411** | **137** | **14031.98** | **10.07** | **Pos. 32 and 33. Probability 0.833392** |
| **LOC_Os11g26880** | **Chr11:‌15444891..15445948(-)** | **1058** | **312** | **104** | **11793.56** | **6.8** | **Pos. 25 and 26. Probability 0.973235** |
| **Glyma.05G151900** | **Chr05:‌34542243..34543022(+)** | **780** | **384** | **128** | **14242.68** | **8.51** | **Pos. 29 and 30. Probability 0.967674** |
| **Glyma.17G026400** | **Chr17:‌1941454..1942617(+)** | **1164** | **360** | **120** | **13086.81** | **8.9** | **Pos. 24 and 25. Probability 0.969189** |
| **Glyma.20G236400** | **Chr20:‌46872743..46878156(+)** | **5414** | **342** | **114** | **12206.91** | **9.04** | **Pos. 29 and 30. Probability 0.653937** |
| **Glyma.05G151400** | **Chr05:‌34512295..34513069(+)** | **775** | **357** | **119** | **13225.96** | **8.61** | **Pos. 27 and 28. Probability 0.648472** |
| **Glyma.03G236000** | **Chr03:‌43592917:‌43593787(-)** | **871** | **522** | **174** | **19727.57** | **9.67** | **Not Signal peptide** |
| **Glyma.19G209900** | **Chr19:‌46430227..46431152(+)** | **926** | **345** | **115** | **12714.66** | **9.05** | **Pos. 25 and 26. Probability 0.915316** |
| **Glyma.13G295800** | **Chr13:‌39511822:‌39512055(+)** | **234** | **198** | **66** | **7339.32** | **10.09** | **Not Signal peptide** |
| **Glyma.10G152000** | **Chr10:‌38682900..38684549(-)** | **1650** | **351** | **117** | **12515.3** | **9.11** | **Pos. 29 and 30. Probability 0.751191** |
| **Glyma.20G066000** | **Chr20:‌23362902..23363123(+)** | **222** | **219** | **73** | **8225.73** | **9.56** | **Pos. 27 and 28. Probability 0.974773** |
| **Glyma.18G002500** | **Chr18:‌223235..224095(-)** | **861** | **366** | **122** | **13897.13** | **8.13** | **Pos. 24 and 25. Probability 0.978834** |
| **Glyma.19G233700** | **Chr19:‌48341606..48342474(-)** | **869** | **387** | **129** | **14404.45** | **8.65** | **Pos. 50 and 51. Probability 0.513865** |
| **Glyma.11G254600** | **Chr11:‌34495373..34496234(+)** | **862** | **366** | **122** | **13858.1** | **8.12** | **Pos. 24 and 25. Probability 0.979290** |
| **Glyma.03G213000** | **Chr03:‌41852186..41853246(+)** | **1061** | **345** | **115** | **12745.57** | **8.61** | **Pos. 25 and 26. Probability 0.823966** |
| **Glyma.11G055200** | **Chr11:‌4163221..4164137(-)** | **917** | **363** | **121** | **13956.88** | **8.67** | **Pos. 24 and 25. Probability 0.675082** |
| **Glyma.08G108700** | **Chr08:‌8344071..8344459(+)** | **389** | **339** | **113** | **12584.75** | **8.59** | **Pos. 29 and 30. Probability 0.976552** |
| **Glyma.15G075300** | **Chr15:‌5774688..5775586(-)** | **899** | **372** | **124** | **13815.5** | **7.7** | **Pos. 22 and 23. Probability 0.976068** |
| **Glyma.07G198100** | **Chr07:‌36668110..36669252(-)** | **1143** | **333** | **111** | **12376.84** | **7.74** | **Pos. 22 and 23. Probability 0.978805** |
| **Glyma.01G186800** | **Chr01:‌52208115:‌52209161(+)** | **1047** | **522** | **174** | **19566.69** | **8.89** | **Not Signal peptide** |
| **Glyma.07G247500** | **Chr07:‌42622460..42623461(-)** | **1002** | **354** | **118** | **12778.56** | **9.08** | **Pos. 23 and 24. Probability 0.960107** |
| **Glyma.13G178300** | **Chr13:‌29206854..29207432(+)** | **579** | **333** | **111** | **12506.05** | **8.8** | **Pos. 22 and 23. Probability 0.978454** |
| **Glyma.08G108200** | **Chr08:‌8317340..8318268(+)** | **929** | **390** | **130** | **14447.3** | **8.08** | **Pos. 27 and 28. Probability 0.906884** |
| **AtRALF20** | **Chr2:‌14691411..14691629(+)** | **219** | **216** | **72** | **7717.12** | **9.1** | **Pos. 27 and 28. Probability 0.975390** |
| **AtRALF13** | **Chr2:‌8251674..8251892(+)** | **219** | **216** | **72** | **7881.11** | **8.61** | **Pos. 17 and 18. Probability 0.980299** |
| **AtRALF11** | **Chr2:‌8247783..8248001(+)** | **219** | **216** | **72** | **7882.14** | **8.61** | **Pos. 17 and 18. Probability 0.980105** |
| **AtRALF2** | **Chr1:‌8203962..8204255(+)** | **294** | **291** | **97** | **11111.23** | **9.97** | **Pos. 25 and 26. Probability 0.973732** |
| **AtRALF12** | **Chr2:‌8249923..8250141(+)** | **219** | **216** | **72** | **7880.11** | **8.7** | **Pos. 17 and 18. Probability 0.978618** |
| **AtRALF32** | **Chr4:‌8092833..8093382(-)** | **550** | **351** | **117** | **12918.62** | **8.19** | **Pos. 26 and 27. Probability 0.976708** |
| **AtRALF24** | **Chr3:‌8586382..8587056(+)** | **675** | **354** | **118** | **13243.29** | **8.32** | **Pos. 22 and 23. Probability 0.943860** |
| **AtRALF30** | **Chr4:‌7624680..7625192(-)** | **513** | **228** | **76** | **8216.81** | **8.99** | **Pos. 22 and 23. Probability 0.978065** |
| **AtRALF7** | **Chr1:‌22392729..22392974(+)** | **246** | **243** | **81** | **9092.57** | **9.59** | **Pos. 25 and 26. Probability 0.907390** |
| **AtRALF5** | **Chr1:‌13049164..13049433(+)** | **270** | **267** | **89** | **10414.38** | **9.63** | **Pos. 25 and 26. Probability 0.972938** |
| **AtRALF3** | **Chr1:‌8205872..8206206(+)** | **335** | **270** | **90** | **10239.91** | **9.5** | **Pos. 29 and 30. Probability 0.970106** |
| **AtRALF6** | **Chr1:‌22333916..22334161(+)** | **246** | **243** | **81** | **9035.4** | **9.42** | **Pos. 29 and 30. Probability 0.975108** |
| **AtRALF10** | **Chr2:‌8246220..8246441(+)** | **222** | **219** | **73** | **7979.46** | **9.67** | **Pos. 17 and 18. Probability 0.971024** |
| **AtRALF21** | **Chr3:‌1292408..1292725(-)** | **318** | **315** | **105** | **11951.04** | **9.8** | **Pos. 30 and 31. Probability 0.958095** |
| **AtRALF29** | **Chr4:‌7037086..7037358(-)** | **273** | **270** | **90** | **10308.26** | **10.05** | **Pos. 25 and 26. Probability 0.979851** |
| **AtRALF16** | **Chr2:‌13931166..13931453(-)** | **288** | **285** | **95** | **10668.27** | **7.64** | **Pos. 29 and 30. Probability 0.976492** |
| **AtRALF25** | **Chr3:‌9163340..9163893(-)** | **554** | **222** | **74** | **8523.19** | **10.17** | **Pos. 22 and 23. Probability 0.980658** |
| **AtRALF28** | **Chr4:‌6984051..6984308(-)** | **258** | **255** | **85** | **9389.97** | **9.19** | **Pos. 31 and 32. Probability 0.967711** |
| **AtRALF26** | **Chr3:‌9165744..9166253(-)** | **510** | **228** | **76** | **8610.11** | **10.31** | **Pos. 22 and 23. Probability 0.977670** |
| **AtRALF8** | **Chr1:‌22714921..22715448(-)** | **528** | **246** | **82** | **8906.44** | **9.55** | **Pos. 28 and 29. Probability 0.971899** |
| **AtRALF9** | **Chr1:‌22717060..22717610(-)** | **551** | **225** | **75** | **8259.7** | **9.55** | **Pos. 28 and 29. Probability 0.975755** |
| **AtRALF15** | **Chr2:‌9379578..9379817(+)** | **240** | **237** | **79** | **8830.37** | **10.04** | **Pos. 28 and 29. Probability 0.974513** |
| **AtRALF27** | **Chr3:‌11671741..11672189(+)** | **449** | **351** | **117** | **12698.4** | **8.23** | **Pos. 27 and 28. Probability 0.975614** |
| **AtRALF31** | **Chr4:‌8058072..8058675(-)** | **604** | **339** | **113** | **12676.48** | **5.01** | **Pos. 21 and 22. Probability 0.979978** |
| **AtRALF22** | **Chr3:‌1591277..1591983(+)** | **707** | **357** | **119** | **13029.69** | **8.6** | **Pos. 23 and 24. Probability 0.980322** |
| **AtRALF33** | **Chr4:‌8984858..8985405(+)** | **548** | **348** | **116** | **12805.64** | **9.41** | **Pos. 27 and 28. Probability 0.945294** |
| **AtRALF17** | **Chr2:‌13951686..13953478(+)** | **1793** | **216** | **72** | **7413.68** | **9.13** | **Pos. 27 and 28. Probability 0.978148** |
| **AtRALF35** | **Chr1:‌22428605..22428844(-)** | **240** | **237** | **79** | **9061.55** | **9.24** | **Pos. 29 and 30. Probability 0.971269** |
| **AtRALF18** | **Chr2:‌14046521..14046832(-)** | **312** | **309** | **103** | **11390.46** | **9.3** | **Pos. 26 and 27. Probability 0.971721** |
| **AtRALF14** | **Chr2:‌8909467..8909772(+)** | **306** | **303** | **101** | **11718.6** | **9.42** | **Pos. 21 and 22. Probability 0.966995** |
| **AtRALF19** | **Chr2:‌14288425..14289225(+)** | **801** | **330** | **110** | **12397.28** | **9.77** | **Pos. 23 and 24. Probability 0.955190** |
| **AtRALF34** | **Chr5:‌26768020..26768846(+)** | **827** | **387** | **129** | **14729.47** | **6.28** | **Pos. 25 and 26. Probability 0.873801** |
| **AtRALF4** | **Chr1:‌9883038..9883870(+)** | **833** | **330** | **110** | **12661.69** | **9.89** | **Pos. 23 and 24. Probability 0.970521** |
| **AT4G14020** | **Chr4:‌8095215..8095736(-)** | **522** | **333** | **111** | **12790.05** | **6.2** | **Not Signal peptide** |
| **AT2G32890** | **Chr2:‌13952716..13954115(-)** | **1400** | **231** | **77** | **8377.42** | **4.19** | **Pos. 26 and 27. Probability 0.975428** |
| **AtRALF1** | **Chr1:‌653765..654343(-)** | **579** | **360** | **120** | **12966.62** | **8.25** | **Pos. 26 and 27. Probability 0.977446** |
| **AtRALF36** | **Chr2:‌13903272..13903487(+)** | **216** | **213** | **71** | **8016.43** | **9.41** | **Pos. 22 and 23. Probability 0.980367** |
| **AtRALF37** | **Chr2:‌13904916..13905110(+)** | **195** | **192** | **64** | **7209.62** | **9.34** | **Pos. 31 and 32. Probability 0.969211** |
| **AtRALF23** | **Chr3:‌5644698..5645414(+)** | **717** | **414** | **138** | **15048.91** | **8.88** | **Pos. 31 and 32. Probability 0.595335** |

Gene basic information, protein basic information and predicted signal peptide cleavage sites in RALF precursor proteins are counted. To identify candidate RALFs from *Oryza sativa*, *Triticum aestivum*, *Brassica rapa*, *Zea mays*, and *Glycine max*, refer to the genome annotation document: Oryza sativa v7.0 (https://phytozome-next.jgi.doe.gov/info/Osativa_v7_0); Triticum aestivum v2.2 (https://phytozome-next.jgi.doe.gov/info/Taestivum_v2_2); Brassica rapa FPsc v1.3 (https://phytozome-next.jgi.doe.gov/info/BrapaFPsc_v1_3); Zea mays RefGen_V4 (https://phytozome-next.jgi.doe.gov/info/Zmays_RefGen_V4); Glycine max Wm82.a2.v1 (https://phytozome-next.jgi.doe.gov/info/Gmax_Wm82_a2_v1). The genes highlighted in red were not considered RALF candidates
